# The impact of horizontal gene transfer in shaping operons and protein interaction networks – direct evidence of preferential attachment

**DOI:** 10.1186/1471-2148-8-23

**Published:** 2008-01-24

**Authors:** Wagied Davids, Zhaolei Zhang

**Affiliations:** 1 Banting & Best Department of Medical Research (BBDMR), Donnelly Centre for Cellular & Biomolecular Research (CCBR), University of Toronto, 160 College Street, Toronto, ON M5S 3E1, Canada; 2 Department of Molecular Genetics, University of Toronto, Toronto, ON M5S 3E1, Canada

## Abstract

**Background:**

Despite the prevalence of horizontal gene transfer (HGT) in bacteria, to this date there were few studies on HGT in the context of gene expression, operons and protein-protein interactions. Using the recently available data set on the *E. coli *protein-protein interaction network, we sought to explore the impact of HGT on genome structure and protein networks.

**Results:**

We classified the *E. coli *genes into three categories based on their evolutionary conservation: a set of 2158 *Core *genes that are shared by all *E. coli *strains, a set of 1044 *Non-core *genes that are strain-specific, and a set of 1053 genes that were putatively acquired by horizontal transfer. We observed a clear correlation between gene expressivity (measured by Codon Adaptation Index), evolutionary rates, and node connectivity between these categories of genes. Specifically, we found the *Core *genes are the most highly expressed and the most slowly evolving, while the *HGT *genes are expressed at the lowest level and evolve at the highest rate. *Core *genes are the most likely and *HGT *genes are the least likely to be member of the operons. In addition, we found the *Core *genes on average are more highly connected than *Non-core *and *HGT *genes in the protein interaction network, however the *HGT *genes displayed a significantly higher mean node degree than the *Core *and *Non-core *genes in the defence COG functional category. Interestingly, *HGT *genes are more likely to be connected to *Core *genes than expected by chance, which suggest a model of differential attachment in the expansion of cellular networks.

**Conclusion:**

Results from our analysis shed light on the mode and mechanism of the integration of horizontally transferred genes into operons and protein interaction networks.

## Background

It is generally accepted that horizontal gene transfer (HGT) is an important process in bacterial genome evolution, which provides both novel metabolic capabilities, and catalyzing the diversification of bacterial lineages [1,2]. Although, the extent of the evolutionary impact of HGT is still under debate [3], it is generally accepted that roughly 10–40% of the protein-coding genes are likely to have been introduced by HGT into the *E. coli *K12 genome [4] since the species divergence from the *Salmonella *lineage approximately 100 million years ago [5].

Currently, no plausible mechanisms have been proposed for the incorporation of HGT genes into their recipient genomes. We envisage that successful incorporation of a horizontally transferred gene needs not only its successful transcription and translation, but also its integration into the existing functional cellular network. We foresee a number of barriers that potentially exist against the incorporation and expression of horizontally transferred genes in a new recipient genome.

The first step of integration for horizontally transferred genes is its incorporation into the host transcription machinery. Bacterial genes are often organized into groups called operons, which enable a simple and unified mechanism of gene regulation in bacteria. Integrating into operons may be regarded as beneficial for the foreign invading genes, since they gain the opportunity not only to be co-regulated and but also co-expressed with resident genes. Secondly, HGT genes may need to optimize their codon usage to be compatible to the host in order to be efficiently transcribed and translated. Thirdly, the protein product has to be integrated into the functional cellular network in order to gain interaction partners and contribute fitness benefits to the organism. Failure to achieve any of the above steps may result in eventual degradation and pseudogenization.

Considering the prevalence of horizontal gene transfer during bacterial genome evolution, the importance of studies exploring their mode of evolution, expression and impact on genomic organization and protein-interactions would thus further our understanding of horizontal gene transfer. With the emergence of high-throughput functional genomics and proteomics data, we are offered a unique opportunity of answering these questions. Thus our specific aims in this paper were to address the following questions:

### (i). Evolutionary Rates and Gene Expression characteristics of Core, Non-core and HGT genes

Bacterial genomes are known to be dynamic, consisting of genes with different evolutionary histories. Some genes are evolutionarily conserved while others can be gained and lost in a lineage-specific fashion, and by horizontal gene transfer events. Prior studies on yeast and vertebrates have suggested that genes that are the most evolutionary conserved and most highly expressed evolve at the slowest rate [6,7]. Therefore to investigate the effect of selection on these various gene categories, we classified *E. coli *genes according to their evolutionary conservation into *Core*, *Non-core *and *HGT *genes (see **Methods**). In this regard, we hypothesize that the cumulative effect of selection acting on these different gene categories would leave footprints in their sequence and gene expression characteristics.

### (ii). The contribution of HGT to operon formation

It is known that horizontally transferred genes can be inserted into existing operons and thus contribute to the dynamic nature of the gene order and membership of these operons [8-15]. Although a few studies have investigated the evolutionary stability and the conservation of gene order of operons [16,17], the relative contribution of HGT on the evolutionary composition of operons remains unclear. In this regard, we aimed to explore the prevalence of *HGT *genes in operons by cataloguing the presence of operons consisting of *Core*, *Non-core *and *HGT *genes.

### (iii). The impact of HGT on protein-protein interactions and networks

Another area that has been missing in the study of HGT events is the aspect of protein-protein interactions and cellular networks. A few studies have concentrated on the impact of horizontal gene transfer on metabolic networks [18,19]. Unfortunately very little is known about the effect of horizontal gene transfer on the global protein interaction networks in this aspect, mostly due to the lack of cellular interaction data in bacteria until recently.

It has been suggested that the scale-free properties of biological networks may in part be due to a model of preferential attachment by means of gene duplication, whereby new nodes preferentially attach to existing highly connected nodes. In networks that have evolved via preferential attachment, older nodes should have a higher average connectivity than younger nodes [20]. In this regard, horizontal gene transfer can be considered as an additional biological mechanism to the existing model of preferential attachment. Although distinctly different, a model of network growth and expansion that involves gene duplication results in a duplicate protein copy with exact same or similar function, whereas a mechanism involving HGT may represent novel functions. In this regard, proteins encoded by *HGT *genes can be seen as competing with resident genes in establishing and gaining protein interactions.

We investigated both operons and protein interactions as a means of detecting successful incorporation of putative horizontally transferred genes in the *E. coli *genome. We explored the possibility that successful *HGT *genes would require integration at the level of operons to be expressed and integration at the network level to establish fitness benefits to the organism. We found horizontally transferred genes exhibit lower gene expressivity and evolve at faster evolutionary rates than evolutionarily conserved core genes. In addition, although proteins encoded by horizontally transferred genes have lower network connectivity, they preferentially attach to resident *Core *proteins rather than *Non-core *proteins within the protein interaction network. We conclude that a small proportion of the low connectivity proteins may have arisen from HGT events.

## Methods

### Data

Genome sequences available for the various *E. coli *strains were downloaded from the NCBI (*Escherichia coli K12 MG1655 *– NC_000913; *Escherichia coli O157H7 *– NC_002695; *Escherichia coli O157H7_EDL933 *– NC_002655; *Escherichia coli CFT073 *– NC_004431; *Escherichia coli UTI89 *– NC_007946).

### Deriving a set of *HGT *genes in *E. coli*

Our primary data set consisted of horizontal gene transfer events that were identified using a combination of the gene phylogeny and the pattern of gene presence and gene absence [15]. This approach is similar to gene presence/absence analyses [21,22].

For detection of horizontal gene transfer events, a total of 326 complete bacterial genome sequences divided into 8 bacterial clades were downloaded from MicrobesOnline database [23]. Using each protein sequence contained within the *E. coli *K12 genome as query, BLASTP sequence similarity searches are conducted against all 326 bacterial proteomes. Subsequent BLAST sequence hits are further categorized into "BestN" hit categories with the Best0 category referring to the *E. coli *K12 gene itself. Each gene is assigned to a relative age category (i.e. clade) based on the BLASTP hit with the highest score. The method classifies each gene within the *E. coli *K12 genome as belonging to either (i) a set of horizontally transferred gene set (named HGT), (ii) a native gene set restricted to the *E. coli *lineage (named Native) or (iii) a gene set with no known sequence homologs (named ORFan). Thus the BLASTP scores gradually decrease in groups with increasing phylogenetic distance from *E. coli *K12.

Multiple sequence alignments based on protein sequences are then constructed using the MUSCLE sequence alignment software [24]. Fast neighbour-joining trees [25] are then subsequently constructed for each protein sequence alignment. Genes that lack "close" homologs in consecutive groups of related bacteria are then confirmed using a quartet test available within the software package TREE-PUZZLE [26]. To infer a horizontal gene transfer event; gene trees are compared with the MicrobesOnline specie tree (see above). If a strongly supported clade in the gene tree was present in disparate genomes, so that three or more deletion events would be required to explain the distribution of the subfamily on the species tree, then an HGT event was assigned.

In addition, we have included a comparison of horizontally transferred genes obtained by various *HGT *detection methods which comprised three surrogate (non-tree based) methods namely, HGT-DB [27], the method published by Mrazek and Karlin [28] and a support vector machine-based method (HGT_SVM) developed by Tsirigos and Rigoutsos [29] versus our data derived from a combined phylogenetic and gene presence/absence based method [15], both in terms of overall counts but also in terms of their distribution of Cluster of Orthologous (COG) functional categories (see Additional File 1).

On the overall, the method developed by Price et al. predicts more *HGT *genes in *E. coli *K12 than the three surrogate methods. It is known that base compositional differences between resident and invading genes are "ameliorated" over a few million years [30]. Surrogate methods that use a compositional approach may preferentially detect recent horizontal gene transfer events and genes with atypical base compositions [21]. Thus, a cross-phylum approach using phylogenetic tree based methods combined with gene phyletic profiles are more likely to detect ancient but also recent horizontal gene transfer events.

### Deriving a set of *Core *and a set of *Non-core E. coli *genes

Our operational definition of a *Core *set of genes was meant to reflect the evolutionary retention of a set of common genes in all *E. coli *strains. In this regard, our *Non-core *set reflect genes that are found in at least one strain but not all strains, and *HGT *genes correspond to genes which are derived from putative recent horizontal gene transfer events. Thus, this distinction between *Core *and *Non-core *genes serves to illustrate the difference between a stable and invariant *Core *component and a variable *Non-core *component that is specific to *E. coli *strains. In this regard, the *Non-core *genes represent genes with a restricted phylogenetic distribution limited to one or more *E. coli *strains. These genes can be lost or gained in a strain-specific manner. Thus to ensure that there is no overlap between any evolutionary gene categories we have filtered the *Core, Non-core *and *HGT *gene sets to ensure a non-overlapping set of each gene category is maintained.

We derived an evolutionary *Core *set of 2158 *E. coli *genes based on the criteria of using phylogenetic gene conservation and genomic context (positional gene conservation). Starting with an all-vs-all protein sequence comparison consisting of the five *E. coli *genomes, we grouped *E. coli *K12 genes based on their phylogenetic gene conservation profiles within all five strains. To ensure a high quality *Core *gene set, we extracted and compared the chromosomal locations of all *Core *genes. It is known that genes which evolve vertically between closely related species can be divided into those that retain homologous chromosomal positions (positional orthologs) and those that do not [31]. In addition, phylogenetic trees were constructed based on selected protein sequences to verify the phylogenetic relationship between the five *E. coli *strains.

Our *Non-core *gene set was obtained by post-process filtering the BLAST sequence comparison results of *E. coli *K12 genes which had BLAST hits in at least one or more *E. coli *genomes, but not present in all genomes. We also extracted and compared gene chromosomal locations of this gene set and constructed phylogenetic trees for further investigation. Since this gene set showed lower phylogenetic conservation, they were also positionally conserved to a lesser extent.

In addition, results from correspondence analysis of codon usage also revealed a distinction between our *Core*, *Non-core *and *HGT *gene categories (Additional File 2). The *Core*,*Non-core *and *HGT *gene lists can be found in Additional Files.

### *E. coli *operons

Data pertaining to *Escherichia coli *operons and transcriptional units were downloaded from RegulonDB version 5.7 [32,33]. RegulonDB is a manually curated database that focuses on transcriptional regulation in *E. coli *with information extracted from literature as well as sequence databases such as GenBank. Its basic structural unit is the operon, which describes the elements and properties of transcriptional regulation. Thus in keeping with this definition, we refer to an operon as a poly-cistronic transcribed unit with its associated regulatory sites, whereas a regulon is defined as a group of operons controlled by a single regulator. As of RegulonDB version 5.7, 4570 *E. coli *genes have been annotated and organized into 2684 operons.

### Analysis of *E. coli *gene expression

*E. coli *K12 MG1655 microarray gene expression data were downloaded from the NCBI GEO microarray database [34]. We selected the GDS2600 data set, which closely approximates growth under normal conditions. This data set contains a time course which monitors the expression of 4405 *E. coli *genes using spotted cDNAs in stationary phase using LB media. Log2-transformed gene expression values were used and we excluded genes with missing data from the analysis. For each gene, mean gene expression values across time points were calculated and used for subsequent analysis.

### Protein-Protein interaction networks

For construction of the *E. coli *interaction network, we extracted the protein-protein interaction data from a recently published mass spectrometry study [35]. We examined this data set carefully to confirm that it was not biased towards particular pathways or functional categories using the KEGG pathways and COG functional classification databases respectively. The whole analysis was also re-performed using the protein interaction data from Arifuzzaman et al. [36] (Additional Files 3 and 4).

### Software

Detection of orthologs was performed using a reciprocal best-hits approach as implemented in the RSD-algorithm [37]. Multiple sequence alignments were constructed from protein sequences using the ClustalW package [38]. Phylogenetic tree reconstructions were performed using the neighbour-joining method [39]. Evolutionary substitution rates were estimated using the CODEML program available from the PAML package[40]. Network analyses were performed using algorithms implemented in the NetworkX package [41] and visualised using PAJEK [42]. Statistical analyses were performed using the R-programming language environment.

## Results and Discussion

### *HGT *genes evolve faster and have lower expression levels

To investigate the selective pressure acting on organizational units, we classified *E. coli *genes according to their evolutionary conservation into three categories, namely, (i). ***Core Set***: a conserved core set of genes that exist in all *E. coli *strains. (ii). ***Non-core Set***: genes that are found in at least one strain but not in all strains, and (iii). ***HGT Set***: genes that are derived from putative recent horizontal gene transfer events after the divergence of *E. coli *and *Salmonella*. By delineating genes according to their evolutionary conservation, we can more clearly identify the evolutionary forces to which the various evolutionary classes of genes are subjected.

Direct measurements of *E. coli *gene expression were obtained from microarray gene expression experiments (see **Methods**). In addition, we have also used the codon adaptation index (CAI) as a proxy for gene expression data, which we referred as "gene expressivity" [43].

**Figure 1:**
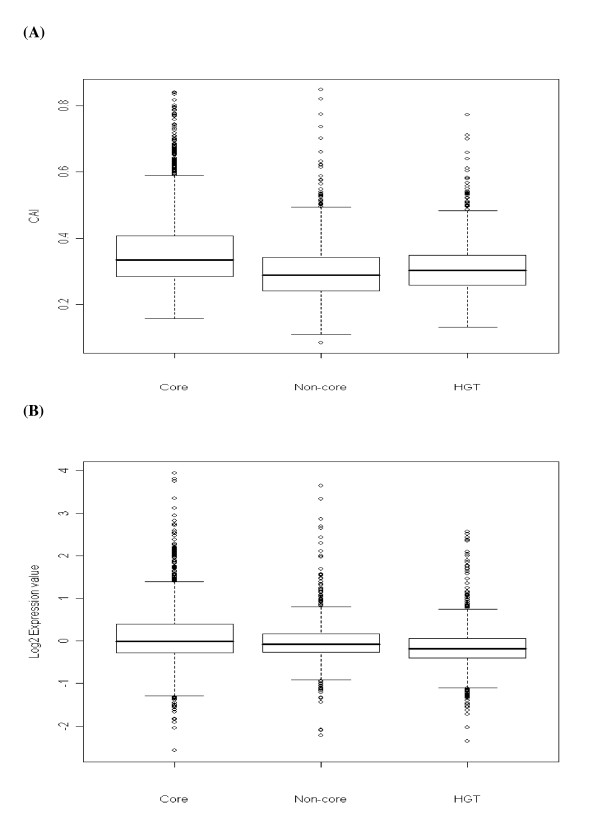
Box plot of **(A) **gene expressivity (CAI) values and **(B) **log2 gene expression values between *Core*, *Non-core *and *HGT *genes. *Core *genes display higher expressivity than *Non-core *and *HGT *genes (P-value < 0.001).

Figure 1 shows that the *Core *genes have higher CAI gene expressivity levels (Figure 1A) as well as log2 expression values (Figure 1B) than *Non-core *and *HGT *genes (t-test and Wilcox rank test, p-value < 0.001). This can be explained by the different evolutionary histories of these three groups of genes. The *Core *set of genes, being the oldest resident genes in the genome have thus had sufficient time to adapt and optimise their codon usage patterns, explaining the higher levels of gene expressivity; whereas the recent horizontally transferred genes may need an adaptation period during which their base composition and codon usage patterns may need to be optimised to their new resident genome.

**Figure 2:**
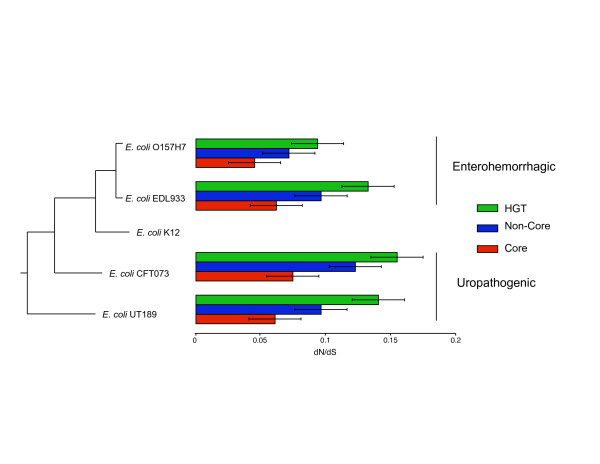
Distribution of evolutionary rates (*dN/dS*) for various *E. coli *strains overlaid on a phylogenetic tree using *E. coli K12 *as reference for genome comparisons. *Core *genes evolve slower than *Non-core *and *HGT *genes (P-value < 0.001).

Figure 2 shows that amongst the three categories of gene sets, the *Core *set of genes evolve at the lowest substitution rates (*dN/dS*) and *HGT *genes evolve at the fastest rates, using *E. coli K12 *as reference for comparison (Wilcoxon-Mann-Whitney test, p-value < 0.001). The high evolutionary rates observed for *HGT *genes may be explained by either one of the following two hypotheses: (i) result of reduced negative selection pressure, which enable the invading genes to be purged from the genome, or (ii) result of increased positive selection whereby *HGT *genes contribute to the phenotypic character of *E. coli *strains [14]. Accordingly, it is thought that the strain-specific *Non-core *genes and *HGT *genes may contribute to the pathogenic character separating the enterohemorrhagic and uropathogenic from the benign *E. coli K12 *strain, therefore these genes are under positive selection pressure.

### Genes in Operons and Networks Display Higher Gene Expressivity

There is increasing evidence to suggest that the chromosomal gene order in organisms is not always random [44]. It is known that proteins of linked genes evolve at comparable rates, and that natural selection may promote the conservation of linkage of co-expressed genes [45]. Accordingly, genes in the same operon occur in close physical proximity and are often known to be co-transcribed as units. In addition, genes encoding subunits of protein complexes also need to be expressed at similar times.

**Figure 3:**
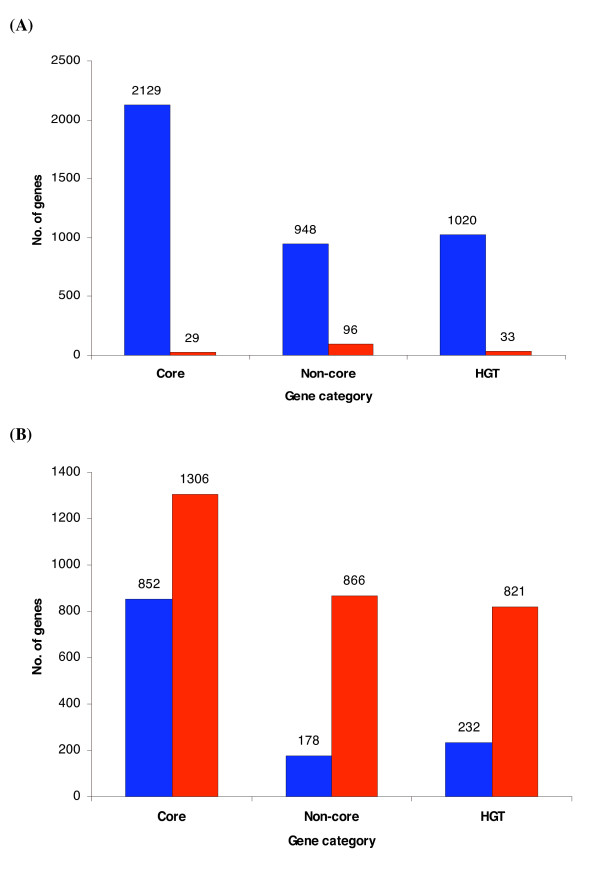
Number of *E. coli *genes in the genome organizations: **(A) **operons, **(B) **protein interaction network (PIN). Genes are classified into three evolutionary categories *Core*, *Non*-*core *and *HGT *genes. *Core *genes predominantly occur in both operons and protein interaction networks (P-value < 0.001).

To investigate the relative contributions of the various evolutionary gene categories on organizational structures, we surveyed both operons and the protein interaction network for their content of *Core*, *Non-core *and *HGT *genes. The *Core *set form a predominant portion of operons with 47% (2129 out of 4506 genes catalogued in RegulonDB version 5.7) of the operons consisting of *Core *genes, whereas 21% (948 out of 4506) of *Non-core *genes and 23% (1020 out of 4506) *HGT *genes, respectively, accounted for the remaining gene constituents of operons (Figure 3A). Similarly, proteins encoded by *Core *genes account for a 67.5% (852 out of 1262) of the protein interaction network as reported by Butland et al [46] whereas *Non-core *genes and *HGT *genes account for 14.1% (178 out of 1262) and 18.4% (232 out of 1262) respectively (Figure 3B).

The tendency of operons to be enriched in *Core *genes can be explained by a need to simplify regulation, since genes residing in operons known to be under control of the same promoter (Chi-squared test, p-value < 0.001). This may facilitate horizontal gene transfer by enabling genes to be inherited as a physical and functional cohesive group rather than separate individual genes. In regard to the protein interaction network, it is thought that the *Core *genes form the ancestral backbones of the protein interaction network to which new functionalities are added via protein nodes and thus strengthens a model by which pathways expand [47].

**Figure 4:**
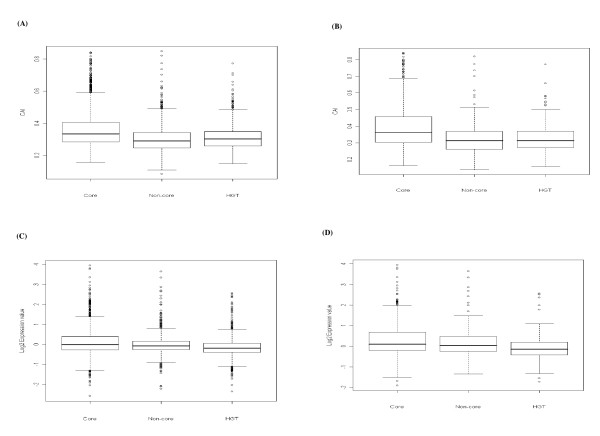
Gene expressivity (CAI) values and log2 gene expression values between *Core*, *Non-core *and *HGT *genes in different genome organizations. **(A) **Box plot of CAI values between *Core*, *Non-core *and *HGT *genes in operons; **(B) **Box plot of gene CAI values between *Core*, *Non-core *and *HGT *genes in protein interaction network (PIN); **(C) **Box plot of log2 gene expression values between *Core*, *Non-core *and *HGT *genes in operons; **(D) **Box plot of log2 gene expression values between *Core*, *Non-core *and *HGT *genes in protein interaction network (PIN).

To understand the impact of higher order organization of genes (i.e. operons) and proteins (i.e. interaction complexes) on properties such as expression or evolution, we investigated the gene expressivity characteristics and evolutionary substitution rates of the various categories of gene sets. We found that *Core *genes in organizational clusters (both operons and protein interaction network or PIN) have higher gene expressivity (CAI) values (Figure 4A and 4B) and as well as log2 expression values (Figure 4C and 4D) relative to *Non-core *and *HGT *genes (t-test and Wilcox-test for both operons and PIN, p-value < 0.001). For the PIN, this trend was robust against removal of ribosomal proteins.

The overall trend from surveying operons and the protein interaction network indicates that *Core *genes tend to be found more often in organizational units such as operons and networks. The evolutionary composition may be the reason that highly clustered proteins in the protein interaction network display apparently high gene expressivity and low substitution rates.

### Distribution of COG Functional Categories between *Core, Non-core *and *HGT *genes within the Operons and Protein Interaction Network

**Figure 5:**
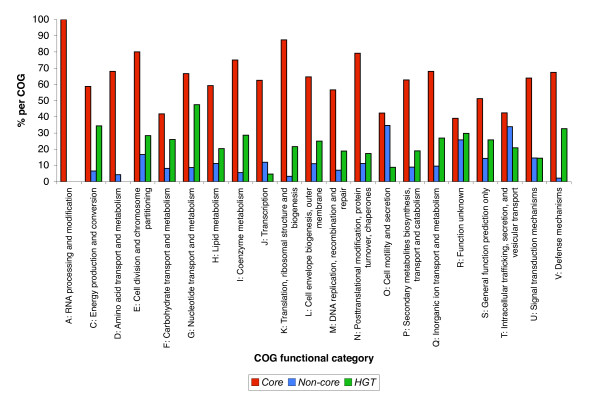
Distribution among the COG categories for all the E. coli genes. Counts were estimated for each evolutionary gene category, and expressed as percentages per total number of genes per COG category. The *Core*, *Non-core *and *HGT *gene sets differ in their distribution of COG functional categories (P-value < 0.001).

**Figure 6:**
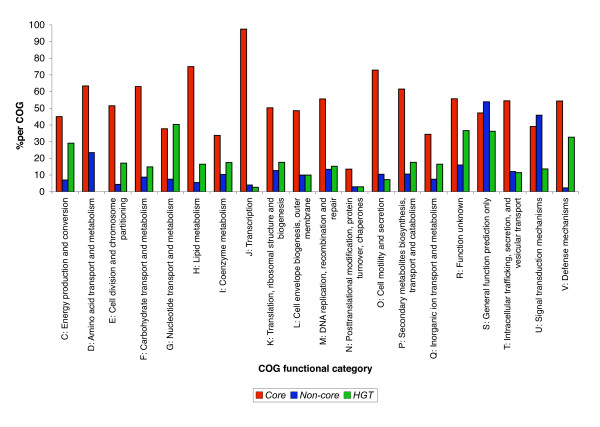
Distribution among the COG categories for those E. coli genes that are members of operons. Counts were estimated for each evolutionary gene category, and expressed as percentages per total number of genes per COG category. The *Core*, *Non-core *and *HGT *gene sets contained within operons differ in their distribution of COG functional categories (P-value < 0.001).

**Figure 7:**
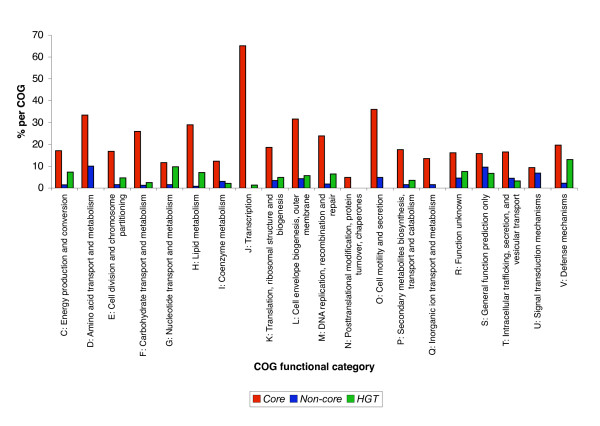
Distribution among the COG categories for those E. coli genes that are members of proten-protein interaction network. Counts were estimated for each evolutionary gene category, and expressed as percentages per total number of genes per COG category. The *Core*, *Non-core *and *HGT *gene sets contained within the protein interaction network differ in their distribution of COG functional categories (P-value < 0.001).

We have analyzed and compared the distribution of the Cluster of Orthologous (COG) functional categories of the *Core*, *Non-core *and *HGT *genes within the *E. coli *K12 genome, protein interaction network and operons (Figures 5, 6 and 7). The various gene categories differ significantly in their COG distribution in the genome, the protein interaction network and operons (Scheirer-Ray-Hare test, p-value < 0.001, see Additional File 5).

In the overall gene comparison of the *E. coli *K12 *Core*, *Non-core *and *HGT *gene sets, the *Core *genes constituted the major evolutionary gene set present in all COG categories (Figure 5). The *Non-core *gene set in comparison to the *HGT *gene set was markedly abundant in the two COG categories: **O **(*Posttranslational modification, protein turnover, chaperones*) and **T **(*Signal transduction mechanisms*). The *HGT *gene set was more abundant than the *Non-core *gene set in the COG categories **C **(*Energy production and conversion*), **F **(*Nucleotide transport and metabolism*), **G **(*Carbohydrate transport and metabolism*), **I **(*Lipid metabolism*), **K **(*Transcription*) and **V **(*Defense mechanisms*). For the operons, the *Core *genes occur predominately in all COG functional categories, whereas the *Non-core genes *are over-represented in COG categories **S **(*Function unknown*) and **U **(*Intracellular trafficking, Secretion, and vesicular transport*) and the *HGT *genes are over-represented in comparison to the *Non-core *genes in COG functional categories **C**, **E **(*Amino acid transport and metabolism*), **G**, **H **(*Coenzyme metabolism*), **R **(*General function prediction only*) and **V **(*Defence mechanisms*) (Figure 6).

For the protein interaction network, the *HGT *genes are over-represented in COG functional categories most notably **C**, **G**, **H**, and **V **(Figure 7). A most notable example in this regard is the COG category **V **in which the *HGT *gene set within the *E. coli *protein interaction network has a significantly higher mean node degree than the *Core *and *Non-core *genes sets. The overall statistical difference in distribution of COG functional categories between the *Core*, *Non-core *and *HGT *gene sets therefore seems to argue against the notion of a *Core*-versus-*Non-core *or *Core*-versus-acquired gene category consisting of *Non-core *and *HGT *genes, but rather strengthens the notion of a distinct separate category for *Non-core *genes.

### Network topology of the *E. coli *genes

To investigate the mode and mechanism of integration of horizontally transferred genes into the *E. coli *protein-protein interaction network, we systemically investigated the network characteristics of proteins encoded by the various evolutionary categories of genes (Table 1). We found that proteins corresponding to the *Core *gene set represent the most highly connected protein nodes, which have an average connectivity of 11.0 interactors (Chi-squared test, p-value < 0.05). In contrast, *Non-core *proteins and proteins encoded by *HGT *genes have on average lower connectivities of 4.0 and 3.0 interactors respectively. This is consistent with our hypothesis that *Core *genes being the most highly conserved genes have resided in the genome for much longer, and thus had more opportunities to evolve interactions. The result of the network analysis is consistent with this theory.

**Table 1:** Protein interaction network characteristics of *E. coli Core, Non-core *and *HGT *genes

Characteristic	*Core*	*Non-core*	*HGT*
Total nodes (1276)	852 (66.8%)	178 (14.0%)	232 (18.2%)
Ave. Node degree	10.9	4.1	3.4
Ave. clustering coefficient	0.100	0.072	0.039
Ave. betweenness centrality	2.59e-3	6.3e-4	5.5e-4

We also analyzed two additional network properties: *betweenness centrality *and *clustering coefficient *(Table 1). *Betweenness centrality *characterizes how essential a node is in maintaining communication between each pair of nodes in a network [48]. Depending on its position within the network, removal of a node can have very different effects on the connectivity, topology and flux of the network. Some nodes can be removed without any harmful effect, while others separate a connected network into disconnected sub-graphs. *Betweeness centrality *is a measure devised to describe the fraction of shortest paths going through a given node, with high values indicating that a node can reach many other nodes. Removal of nodes with high centrality will make it difficult to reach from one node to another, thus lengthen the path between nodes. The *clustering coefficient *describes the local transitivity in a network, with two nodes having a common neighbour in a network being more likely to be neighbours [49].

Table 1 shows that the *HGT *genes have lower *betweenness centrality *than the *Core *and *Non-core *genes, which suggests that they are less important in cellular communications. Interestingly the *Non-core *genes have higher *betweeness centrality *than the *Core *genes, the implication of which need to be further explored. On the other hand, *Core *genes have the highest *clustering coefficients*, with any two *Core *genes having a common neighbour being more likely to be neighbours of each other. The results in Table 1 indicate the *HGT *genes are the least important in maintaining the overall connectivity of the protein interaction network, in other words they are more likely to be *peripheral nodes*.

Our analysis of the distribution of COG functional categories of the *Core*, *Non-core *and *HGT *nodes within the *E. coli *protein interaction network reveal that the *Core *genes are the most abundant and cover all the major COG functional categories in comparison to the *Non-core *and *HGT *gene sets (Figure 7). Although, the *Non-core *and *HGT *genes show similar COG distribution profiles within the protein interaction network, differences exist in COG categories **C**, G, **H **and **V **in which the *HGT *genes are relatively more abundant than *Non-core *genes. A most notable result in this regard is the COG defense category (**V**) in which the *HGT *gene set within the *E. coli *protein interaction network has a significantly higher mean node degree than the *Core *and *Non-core *genes set.

### Preferential Attachment of HGT proteins to *Core *proteins

We further investigated the evolutionary profiles of the interaction partners in the network. Table 2 shows that about 74% of all the interactions are between a pair of *Core *genes, 11.2 % of the interactions are between a *Core *gene and a *Non-core *gene. In other words, in total about 85% of the interactions involve at least one *Core *gene. Among the interactions involving *HGT *genes, a large percentage (89%) was between a *HGT *genes and a *Core *gene, while interactions between *Non-core *and *HGT *genes only account for 1%. This is surprising since the ratio between *Core *genes and *Non-core *genes is only ~5:1, much smaller than the 9:1 ratio (89%: 10%) that we observed in the network. This discrepancy in ratio implies that an *HGT *gene have a higher propensity to establish interaction with a *Core *gene than with a *Non-core *gene. Indeed, the proportions of HGT-Core interactions are higher than expected by chance (Chi-squared test, p-value < 0.001).

**Table 2:** Classification of interactions based on the evolutionary profiles of interaction partners.

Category of interacting partners	Number of Interactions
*Core to Core*	3981 (74.0%)
*Non-core to Non-core*	35 (0.6%)
*HGT to HGT*	24 (0.4%)
*Core to Non-core*	606 (11.2%)
*Core to HGT*	687 (12.8%)
*Non-core to HGT*	55 (1.0%)
Total Interactions	5388 (100%)

Such a model of preferential attachment has previously been proposed to explain the growth of protein interaction networks in *S. cerevisiae *[20,50,51] and was also suggested recently for *E. coli *[52]; however it has remained mostly unproven since it was difficult to trace back the evolution history of protein networks. Along this line, the *HGT *genes in *E. coli *offer a unique opportunity to test this theory since these genes are indeed "new genes" that were only added to the network after the HGT event ~100 millions ago [5]. Our observation provided direct evidence and support for this model, which has not been reported previously.

#### Data Availability 

Additional file 6 contains the data used and produced in this study. 

## Conclusion

To our knowledge, our analysis represent the first time that the HGT events are investigated in the context of protein-protein interaction and cellular networks. This is important because horizontal gene transfer in known to be prevalent in bacterial genome evolution in shaping the genome content, and they had an impact on the stability and evolution of the protein interactions and network.

From our analyses, the distinguishing characteristics which sets the *HGT *gene category apart from the *Non-core *and *Core *gene categories are (i) higher evolutionary substitution rates (*Ka/Ks*), (ii) protein interaction network statistical properties such as protein *degree connectivity, average clustering coefficients and betweeness centrality*, (iii) preferential attachment with regards to the number of interactions formed by *HGT *genes, which indicate that *HGT *proteins preferentially neither self-associate nor do *HGT *proteins associate with *Non-core *proteins within the *E. coli *protein interaction network.

**Figure 8:**
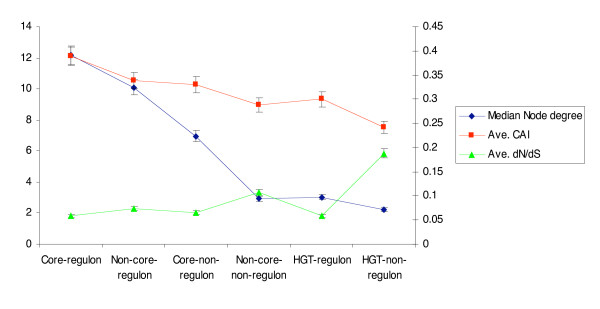
Summary of the relationship between protein connectivity, gene expressivity (CAI) and evolutionary rates (*dN/dS*) in *E. coli*.

Results from our study revealed a clear relationship between gene expressivity, evolutionary rate and protein connectivity for the three evolutionary classes of genes (Figure 8). The conserved *Core *set of genes generally display higher gene expressivity and protein connectivity than strain-specific *Non-core *and *HGT *genes. However, both gene expressivity and protein connectivity are inversely related to evolutionary rates, with the most highly conserved genes evolving the slowest. In contrast, horizontally transferred genes evolve at considerably higher evolutionary rates, and have lower gene expressivity and protein connectivity. In addition, proteins encoded by horizontally transferred genes attach preferentially to *Core *proteins within the *E. coli *protein interaction network. Consistent with this finding is the general idea that *Core *genes are the oldest resident genes and form the backbone of the protein interaction network to which new proteins are attached. These results may also suggest that a proportion of the lowest connectivity proteins in bacterial protein interaction networks are those genes which are more likely to have recently been transferred and incorporated into the *E. coli *genome.

This is reminiscent of the so-called "Complexity Hypothesis", which was proposed to explain why the successful horizontal transfer of a gene is less probable if the connectivity of the protein it encodes is large [52], and its later modification called the 'Extended Complexity hypothesis' [53] which aims to explain why adaptive evolution is the least likely for proteins with high complexity. Although the Complexity Hypothesis and its modified version aim to describe which types of genes are more or less likely to be subjected to horizontal gene transfer, it fails to provide a mode and mechanism for subsequent integration of the horizontally transferred gene into it new recipient genome. The results from our analysis support these hypotheses with genomics and evolutionary data.

Considering the prevalence of HGT in bacteria, the relative contribution of HGT as an additional mechanism to gene duplication may become more important on network evolution. Thus, with the availability of proteomics data for more bacteria, we will most likely gain more insight on the impact of HGT on the evolution of networks.

## List of abbreviation used

HGT: Horizontal Gene Transfer

PIN: Protein Interaction Network

CAI: Codon Adaption Index

BLAST: Basic Local Alignment and Search Tool

KEGG: Kyoto Encyclopedia of Genes and Genomes http://www.genome.jp/kegg

COG: clusters of orthologous groups http://www.ncbi.nih.gov/COG

## Authors' contributions

WD collected data, carried out the calculations, performed statistical analyses, and drafted the manuscript. WD and ZZ designed the study. ZZ participated in writing the manuscript. All authors read and approved the final manuscript.

## Supplementary Material

Additional file 1 Comparison between different HFT gene detection methods. (A) This is a 4-way comparison Venn diagram illustrating the intersection and differences between various horizontal gene transfer detection methods investigated. The comparison included a non-surrogate phylogeny and gene presence/absence based method developed by Price [15] versus three surrogate methods which included HGT-DB [27], the method published by Mrazek and Karlin [28] and a support vector machine-based method (HGT_SVM) developed by Tsirigos and Rigoutsos [29]. (B): This is a comparison of Cluster of Orthologous Group (COG) functional categories between *Core*, *Non-core *and *HGT *gene sets obtained using various methods of horizontal gene transfer detection. The comparison included a non-surrogate phylogeny and gene presence/absence based method developed by Price [15] versus three surrogate methods which included HGT-DB [27], the method published by Mrazek and Karlin [28] and a support vector machine-based method (HGT_SVM) developed by Tsirigos and Rigoutsos[29]. Click here for file

Additional file 2 Codon usages between *core*, *Non-core *and *HGT *genes. This is a correspondence analysis of codon usage from *E. coli Core*, *Non-core*, and putative *HGT *genes using the first two principal components. Click here for file

Additional file 3 Comparison between two E. coli interaction studies. This is a comparison of COG functional classes between Arifuzzaman et al. (2006) and Butland et al (2005) *E. coli *protein interaction networks. Click here for file

Additional file 4 Comparison between two E. coli interaction studies. This is a comparison between Arifuzzaman et al. (2006) and Butland et al. (2005) published protein interaction data sets. Click here for file

Additional file 5 Statistical tests for the COG distribution. (A) Kruskal-Wallis ANOVA with Scheirer-Ray-Hare extension on the ranks of COG category counts in the Genome. (B) Kruskal-Wallis ANOVA with Scheirer-Ray-Hare extension on the ranks of COG category counts in the Operons. (C) Kruskal-Wallis ANOVA with Scheirer-Ray-Hare extension on the ranks of COG category counts in the protein interaction network (PPI). Click here for file

Additional file 6 Data_2008_0117.zip. Compressed zip file containing data used in the study Click here for file
