# Supplementary material for: The impact of horizontal gene transfer in shaping operons and protein interaction networks – direct evidence of preferential attachment
Source: BMC Evol Biol. 2008 Jan 24;8:23. doi: 10.1186/1471-2148-8-23 (PMC2259305; doi:10.1186/1471-2148-8-23)
Supplement: Additional file 1 — Comparison between different HFT gene detection methods. (A) This is a 4-way comparison Venn diagram illustrating the intersection and differences between various horizontal gene transfer detection methods investigated. The comparison included a non-surrogate phylogeny and gene presence/absence based method developed by Price [15] versus three surrogate methods which included HGT-DB [27], the method published by Mrazek and Karlin [28] and a support vector machine-based method (HGT_SVM) developed by Tsirigos and Rigoutsos [29]. (B): This is a comparison of Cluster of Orthologous Group (COG) functional categories between Core, Non-core and HGT gene sets obtained using various methods of horizontal gene transfer detection. The comparison included a non-surrogate phylogeny and gene presence/absence based method developed by Price [15] versus three surrogate methods which included HGT-DB [27], the method published by Mrazek and Karlin [28] and a support vector machine-based method (HGT_SVM) developed by Tsirigos and Rigoutsos[29]. [file 1471-2148-8-23-S1.pdf]

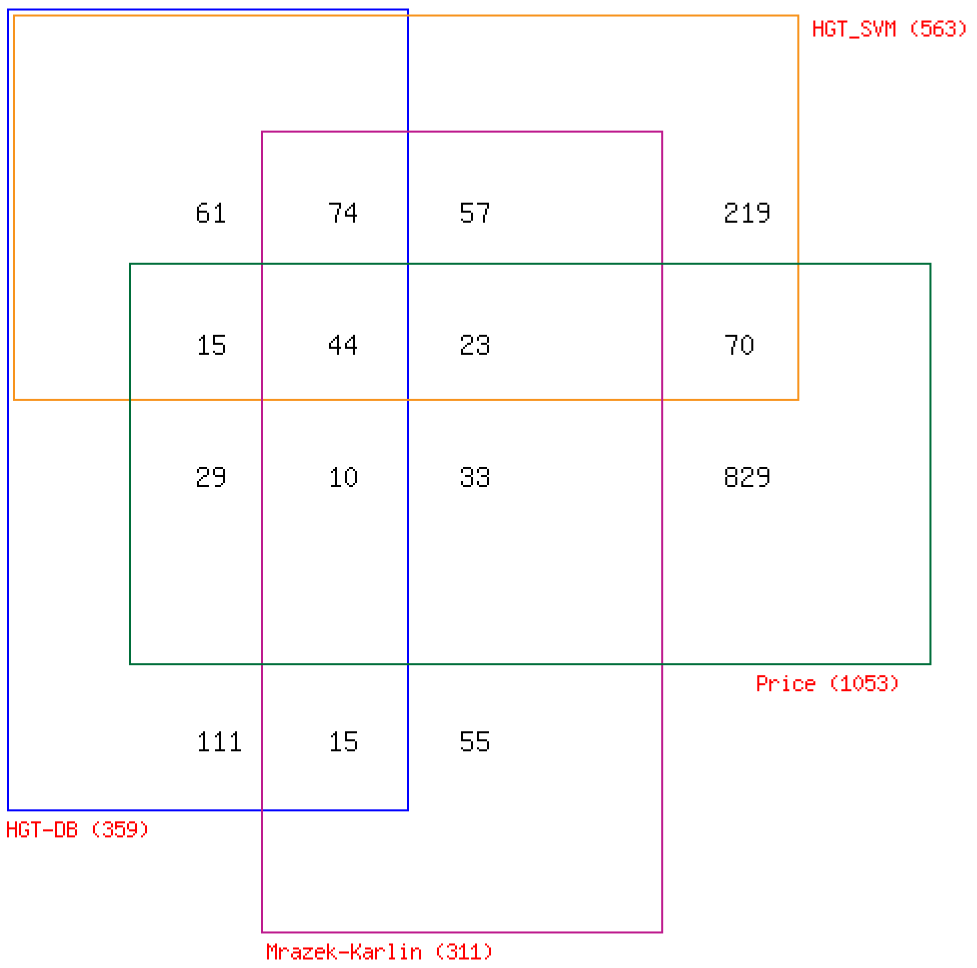

Figure S1-A. 4-way comparison Venn diagram illustrating the intersection and differences between various horizontal gene transfer detection methods investigated. The comparison included a non-surrogate phylogeny and gene presence/absence based method developed by Price (Price et al., 2007) versus three surrogate methods which included HGT-DB (Garcia-Vallve et al., 2003), the method published by Mrazek and Karlin (Mrázek and Karlin, 1999) and a support vector machine-based method (HGT_SVM) developed by Tsirigos and Rigoutsos (Tsirigos and Rigoutsos, 2005).

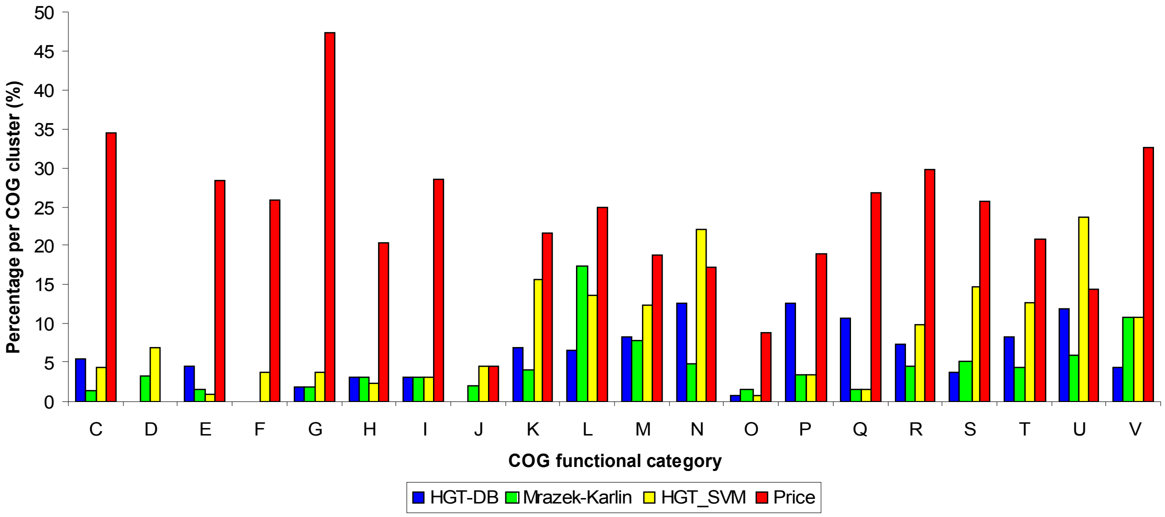

Figure S1-B. Comparison of Cluster of Orthologous Group (COG) functional categories between *Core*, *Non-core* and *HGT* gene sets obtained using various methods of horizontal gene transfer detection. The comparison included a non-surrogate phylogeny and gene presence/absence based method developed by Price (Price et al., 2007) versus three surrogate methods which included HGT-DB (Garcia-Vallve et al., 2003), the method published by Mrazek and Karlin (Mrázek and Karlin, 1999) and a support vector machine-based method (HGT_SVM) developed by Tsirigos and Rigoutsos (Tsirigos and Rigoutsos, 2005).

Phylogeny based gene presence/absence method:
Price- data provide kindly by Morgan Price via personal communication.

Surrogate methods:
HGT-DB - (Garcia-Vallve et al., 2003) Horizontal gene data set taken from HGT-DB (http://www.tinet.org/~debb/HGT/ecoli.d/HGTList.html)
Mrazek-Karlin – (Mrázek and Karlin, 1999)
http://www.cmbl.uga.edu/software/phxpa.html
HGT_SVM - (Tsirigos and Rigoutsos, 2005)
http://cbcsrv.watson.ibm.com/HGT_SVM/predictions.tgz

References
Price M.N, Dehal P.S, and Arkin A.P. 2007 Orthologous transcription factors in bacteria have different functions and regulate different genes. PLoS Comput Biol. 7;3(9):e175.
Garcia-Vallve, S., E. Guzman, M. A. Montero, and A. Romeu. 2003. HGT-DB: a database of putative horizontally transferred genes in prokaryotic complete genomes. Nucleic Acids Res 31:187-189.
Mrázek J and Karlin S. 1999 Detecting alien genes in bacterial genomes. Ann N Y Acad Sci. 870:314-29.
Tsirigos A. and Rigoutsos I. 2005 A sensitive, support-vector-machine method for the detection of horizontal gene transfers in viral, archaeal and bacterial genomes. Nucleic Acids Res. 33(12):3699-3707.
