# Supplementary material for: The impact of horizontal gene transfer in shaping operons and protein interaction networks – direct evidence of preferential attachment
Source: BMC Evol Biol. 2008 Jan 24;8:23. doi: 10.1186/1471-2148-8-23 (PMC2259305; doi:10.1186/1471-2148-8-23)
Supplement: Additional file 2 — Codon usages between core, Non-core and HGT genes. This is a correspondence analysis of codon usage from E. coli Core, Non-core, and putative HGT genes using the first two principal components. [file 1471-2148-8-23-S2.pdf]

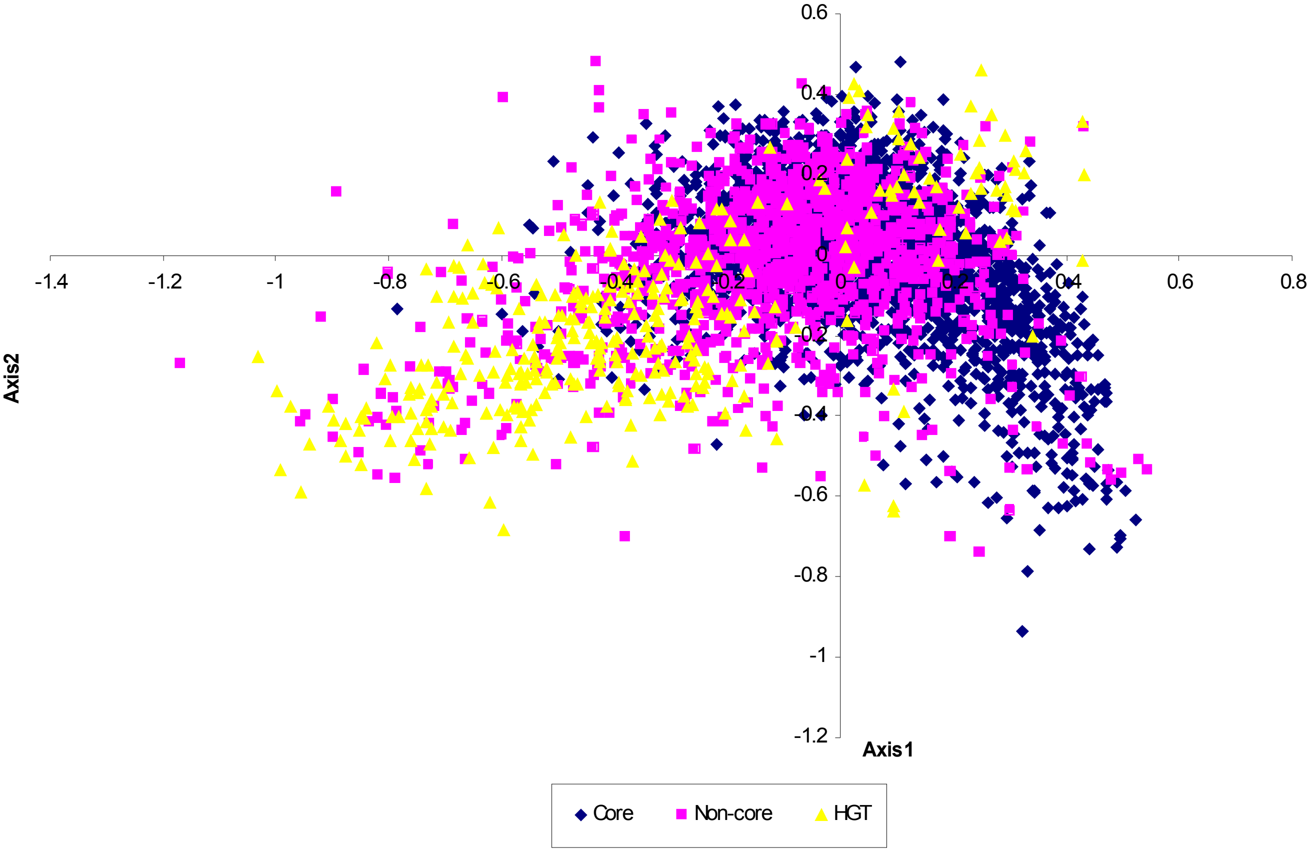

Figure S2. Correspondence analysis of codon usage from *E. coli Core*, *Non-core*, and putative *HGT* genes using the first two principal components.
