# Supplementary material for: The impact of horizontal gene transfer in shaping operons and protein interaction networks – direct evidence of preferential attachment
Source: BMC Evol Biol. 2008 Jan 24;8:23. doi: 10.1186/1471-2148-8-23 (PMC2259305; doi:10.1186/1471-2148-8-23)
Supplement: Additional file 3 — Comparison between two E. coli interaction studies. This is a comparison of COG functional classes between Arifuzzaman et al. (2006) and Butland et al (2005) E. coli protein interaction networks. [file 1471-2148-8-23-S3.pdf]

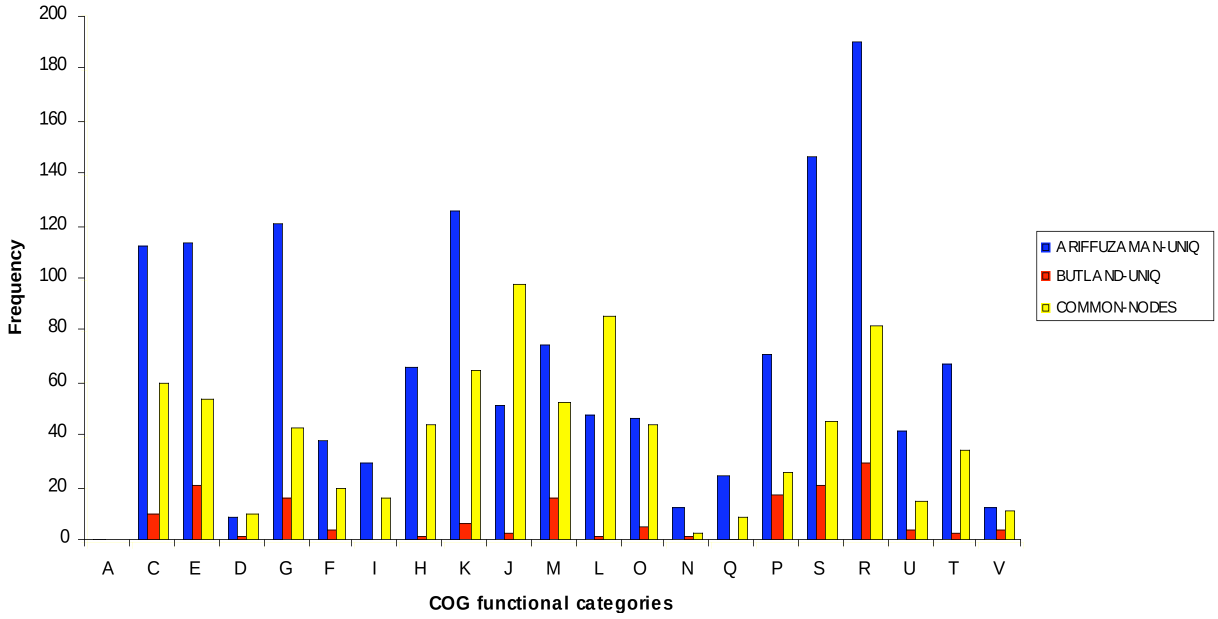

Figure S3. Comparison of COG functional classes between Arifuzzaman et al. (2006) and Butland et al (2005) *E. coli* protein interaction networks.
