# Supplementary material for: The impact of horizontal gene transfer in shaping operons and protein interaction networks – direct evidence of preferential attachment
Source: BMC Evol Biol. 2008 Jan 24;8:23. doi: 10.1186/1471-2148-8-23 (PMC2259305; doi:10.1186/1471-2148-8-23)
Supplement: Additional file 4 — Comparison between two E. coli interaction studies. This is a comparison between Arifuzzaman et al. (2006) and Butland et al. (2005) published protein interaction data sets. [file 1471-2148-8-23-S4.pdf]

COGs	ARIFFUZAMAN-UNIQ	BUTLAND-UNIQ	COMMON-NODES	Tot. no. per COG	COG Functional Groups
A	1	0	0	1	RNA processing and modification
C	113	11	60	276	Energy production and conversion
E	114	21	54	328	Amino acid transport and metabolism
D	10	2	11	30	Cell division and chromosome partitioning
G	121	17	44	268	Carbohydrate transport and metabolism
F	39	4	20	81	Nucleotide transport and metabolism
I	30	0	17	98	Lipid metabolism
H	66	2	45	128	Coenzyme metabolism
K	126	7	65	269	Transcription
J	52	3	98	152	Translation, ribosomal structure and biogenesis
M	75	17	53	218	Cell envelope biogenesis, outer membrane
L	48	2	86	212	DNA replication, recombination and repair
O	47	6	45	125	Posttranslational modification, protein turnover, chaperones
N	13	2	3	104	Cell motility and secretion
Q	25	1	9	67	Secondary metabolites biosynthesis, transport and catabolism
P	72	18	27	200	Inorganic ion transport and metabolism
S	147	21	46	210	Function unknown
R	190	30	82	397	General function prediction only
U	42	4	15	118	Intracellular trafficking, secretion, and vesicular transport
T	68	3	35	158	Signal transduction mechanisms
V	13	4	12	46	Defense mechanisms
Total	1412	175	827	3486	

Table S1. COG Comparison between Arifuzzaman et al. (2006) and Butland et al. (2005) published protein interaction data sets.
