# Supplementary material for: The impact of horizontal gene transfer in shaping operons and protein interaction networks – direct evidence of preferential attachment
Source: BMC Evol Biol. 2008 Jan 24;8:23. doi: 10.1186/1471-2148-8-23 (PMC2259305; doi:10.1186/1471-2148-8-23)
Supplement: Additional file 5 — Statistical tests for the COG distribution. (A) Kruskal-Wallis ANOVA with Scheirer-Ray-Hare extension on the ranks of COG category counts in the Genome. (B) Kruskal-Wallis ANOVA with Scheirer-Ray-Hare extension on the ranks of COG category counts in the Operons. (C) Kruskal-Wallis ANOVA with Scheirer-Ray-Hare extension on the ranks of COG category counts in the protein interaction network (PPI). [file 1471-2148-8-23-S5.pdf]

Table S2A. Kruskal-Wallis ANOVA with Scheirer-Ray-Hare extension on the ranks of COG category counts in the Genome.

	d.f	Sum Sq.	Mean Sq.	H	P-value
Gene category	2	8290.9	4145.4	64.8	8.66e-15 ***
COG category	20	9191.8	459.6	7.2	1.0
Gene:COG	40	3337.8	83.4	1.3	1.0

*** Statistically significant

Table S2B. Kruskal-Wallis ANOVA with Scheirer-Ray-Hare extension on the ranks of COG category counts in the Operons.

	d.f	Sum Sq.	Mean Sq.	H	P-value
Gene category	2	6171.6	3085.8	50.6	1.036e-11 ***
COG category	19	8950.5	471.1	7.7	1.0
Gene:COG	38	2862.4	75.3	1.2	1.0

*** Statistically significant

Table S2C. Kruskal-Wallis ANOVA with Scheirer-Ray-Hare extension on the ranks of COG category counts in the protein interaction network (PPI).

	d.f	Sum Sq.	Mean Sq.	H	P-value
Gene category	2	8152.3	4076.1	66.8	3.109e-15 ***
COG category	19	6656.8	350.4	5.7	1.0
Gene:COG	38	3131.9	82.4	1.4	1.0

*** Statistically significant
