# Supplementary material for: The impact of horizontal gene transfer in shaping operons and protein interaction networks – direct evidence of preferential attachment
Source: BMC Evol Biol. 2008 Jan 24;8:23. doi: 10.1186/1471-2148-8-23 (PMC2259305; doi:10.1186/1471-2148-8-23)
Supplement: Additional file 6 — Data_2008_0117.zip. Compressed zip file containing data used in the study [file 1471-2148-8-23-S6.zip › DATA_2008_0117/PRICE_MANUSCRIPT_2007/TFHist.pdf]

Title: Horizontal gene transfer and the evolution of transcriptional regulation in Escherichia coli

Authors: Morgan N. Price and Paramvir S. Dehal and Adam P. Arkin

Abstract:

We examined the evolutionary histories of transcription factors (TFs) and of regulatory interactions from *Escherichia coli* K12. We show that although most TFs have paralogs, these usually arose by horizontal gene transfer (HGT) rather than by duplication within the *E. coli* lineage. Most global regulators evolved vertically within the γ-Proteobacteria, but most neighbor regulators – TFs that are adjacent to genes that they regulate – were acquired by HGT. Furthermore, neighbor regulators were often acquired together with the adjacent operon that they regulate. Many of the as-yet-uncharacterized TFs have also been acquired together with adjacent genes, so we predict that these are neighbor regulators as well. When we analyzed the histories of regulatory interactions, we found that the evolution of regulation by duplication was rare, and surprisingly, many of the regulatory interactions that are shared between paralogs result from convergent evolution. Another surprise was that HGT genes are more likely than native genes to be regulated by multiple TFs, and most of this complex regulation probably evolved after the transfer. Our results highlight the rapid evolution of niche-specific gene regulation in bacteria.

Introduction

Transcription factors bind to specific sites on DNA where they regulate the expression of target genes and thus allow bacteria to adapt to a changing environment. In the well-studied bacterium *Escherichia coli* K12, over 150 transcription factors have been characterized (Salgado et al., 2006), and nearly 100 more are predicted from the genome sequence. Most of the *E. coli* transcription factors (TFs) include a DNA-binding domain that determines target site specificity as well as a sensing domain that binds to small metabolites or to signaling proteins (Babu and Teichmann, 2003). With the availability of complete genome sequences from diverse bacteria, researchers have begun to ask how these transcriptional factors and their binding sites has evolved (Rajewsky et al., 2002; Babu and Teichmann, 2003; Teichmann and Babu, 2004; Lozada-Chavez et al., 2006; Gelfand, 2006).

Because *E. coli* TFs form large gene families, it appears that most of them have arisen by gene duplication (Otsuka et al., 1996; Babu and Teichmann, 2003). Because two TFs from any given family usually regulate distinct genes and bind to distinct effectors, the duplicates generally have distinct rather than overlapping functions. However, it has not been clear from previous studies whether the duplicates arose within the *E. coli* lineage or were acquired by horizontal gene transfer (HGT), or how long ago these duplication events occurred. For example, the ancestral TF might have been transferred to another lineage, where it diverged and acquired a new function, and could then have been reacquired, to give paralogs that arose by HGT rather than by duplication within the *E. coli* lineage (Gogarten et al., 2002).

The evolution of the regulatory sites that TFs bind to has been studied by comparing upstream sequences across *E. coli* and its relatives (Rajewsky et al., 2002; McCue et al., 2001, 2002). It appears that regulatory sites are usually conserved in close relatives within the family of Enterobacteria, such as *Salmonella typhimurium* and *Klebsiella pneumoniae*, and are often conserved in moderately distant relatives within the γ-Proteobacterial division, such as *Vibrio cholerae* or *Shewanella oneidensis*, as well. So, many of these regulatory sites are quite old (Rajewsky et al., 2002; McCue et al., 2001, 2002). This also implies that these regulatory sites are under strong purifying (negative) selection.

However, because these studies compared orthologous genes in *E. coli* and its relatives, they did not examine the regulation of recently acquired genes. Because most of the genes in *E. coli* K12 were acquired by HGT after the divergence of the γ-Proteobacteria (Lerat et al., 2005), it is important to ask how acquired genes are regulated. HGT genes might evolve new regulation after they are acquired, either because the genes' regulators from the source bacterium are not present in the new host, or because different conditions in the new host select for different regulation. On the other hand, newly acquired genes might be more likely to be fixed in the population if they already contain regulatory sequences that can function in their new host.

It has also been proposed that gene duplication is a major source of regulatory interactions (Teichmann and Babu, 2004). Between 7% (Babu and Teichmann, 2003) and 38% (Teichmann and Babu, 2004) of the regulation in *E. coli* is reported to have arisen by gene duplication, although another group reported that this is rare (Otsuka et al., 1996). Also, about a third of paralogous genes are reported to have conserved operon structure (Janga and Moreno-Hagelsieb, 2004) and conserved regulatory sequences (Rajewsky et al., 2002). Because these studies did not examine whether the paralogs were closely related and whether the regulation was conserved from an ancestral state, these regulatory similarities could reflect convergent evolution instead of conservation from the common ancestors of the genes.

Finally, it has been observed that many of the regulators in *E. coli* are adjacent to operons that they regulate (Hershberg et al., 2005). These "neighbor regulators" usually regulate just one or two operons, and the proximity of these regulators to their regulated genes suggests that horizontal gene transfer might be involved in the evolution of these regulatory relationships (Hershberg et al., 2005). Furthermore, these neighbor regulators are often conserved adjacent to their targets in other genomes (Korbel et al., 2004). However, as far as we know, there has not been a direct test of whether neighbor regulation is associated with HGT.

To clarify the origins of transcriptional regulation in *E. coli*, we performed a detailed phylogenetic analysis of its transcription factors. We found that relatively few of the TFs evolved by duplications within the *E. coli* lineage. Instead, we found a surprisingly complex history of HGT for many of the regulators, especially for the neighbor regulators and the as-yet-uncharacterized regulators. Furthermore, the HGT of these specific regulators together with their regulated genes often allows us to predict regulatory targets. In contrast, most of the global regulators seem to have ancient origins in the γ-Proteobacteria.

We then analyzed the histories of individual regulatory interactions. By comparing the age of the interactions to the age of the genes, we show that few of the regulatory interactions evolve by duplication, but there is a tendency towards convergent evolution of regulatory interactions involving paralogs, both for paralogs of TFs and for paralogs of regulated genes. Also, because global regulators account for about half of all known regulatory interactions, we wondered how they relate to HGT genes. We found that HGT genes tend to be under more complex regulation than of native genes, and the global regulator CRP regulates a higher proportion of HGT genes than native genes. We identified cases where regulatory sites for conserved global regulators have been conserved across HGT events within the γ-Proteobacteria, but most of the regulation of these HGT genes appears to have evolved after the transfer event. This illustrates that major parts of the regulatory network evolved recently under selection. Overall, most of the TFs have been acquired recently, and even for the global regulators, most of their binding sites have evolved relatively recently. We give a schematic overview of our results in Figure 1.

Results

Evolutionary Histories of Transcription Factors

Because most TFs belong to large families and have paralogs, we built phylogenetic trees for the TFs (see Methods) and we manually compared these trees to the species tree shown in Figure 2. We focused on the period after the divergence of *E. coli* from *Shewanella*, as we found phylogenetic reconstruction deeper within the γ-Proteobacteria to be impractical. (Most gene trees are poorly resolved beyond this distance, probably because the phylogenetic signal is reduced once the sequence divergence becomes too great.) According to our species tree (see Methods), this period comprises about a third of *E. coli*'s evolutionary history since the divergence of the bacteria, or perhaps 1 billion years. As we will see, much as changed during this time.

We classified a TF as being acquired by HGT after this divergence if close relatives of the TF were found in more distantly related bacteria, so that three or more gene loss events would otherwise be required to reconcile the gene tree with the species tree (e.g. Figure 3; see Methods for details). We classified a TF as being duplicated within the *E. coli* lineage if it had a paralog that was closely related in the gene tree (e.g., Figure 4). We classified a gene as an "ORFan" if it had no homologs in organisms more distantly related than *Shewanella*. The origin of microbial ORFans is unclear (Yin and Fischer, 2006), but they might be HGT from an unknown source. Finally, we classified other TFs as native (evolving by vertical descent, e.g. Figure 5). However, because our criteria for identifying HGT was conservative, there may be undetected HGT events within the "native" TFs, as well as ancient HGT before the divergence of *E. coli* from *Shewanella*.

Besides phylogeny, we also classified TFs by their function. We analyzed characterized transcription factors from RegulonDB 5.6 (Shen-Orr et al., 2002). We classified the 20 TFs that regulated the

largest number of genes as global regulators. We classified TFs that regulate adjacent genes as neighbor regulators. To exclude auto-regulation, which is common, we classified TFs as neighbor regulators only if they regulate adjacent yet distinct transcription units. (Five of the global regulators also regulate adjacent genes – those were excluded from the neighbor regulators.) We also considered other characterized TFs and putative, as-yet-uncharacterized regulators. We analyzed the history of each of the global regulators, and of a sample of each of the other types of regulators (see Figure 6 and Methods).

Whereas most global regulators were native genes within the γ-Proteobacteria, most neighbor regulators have been acquired after the divergence between *E. coli* and *Shewanella* (Figure 6; the classification for each TF is given in Supplementary Note 1). Other characterized regulators were native, HGT, or duplications within the lineage leading to *E. coli*, in roughly equal proportions. Finally, most of the putative regulators were acquired by HGT (Figure 6). We found little duplication of TFs within the *E. coli* lineage. In the following sections, we examine in more detail the global regulators, the neighbor regulators, and the pattern of HGT.

Vertical evolution of most global regulators

We found that 17 of the 20 global regulators have evolved vertically since the divergence of *E. coli* from *Shewanella*. For example, as shown in Figure 5, crp has mostly evolved vertically, with no evidence for gene gain, and with gene losses only in the highly reduced genomes of the insect endosymbionts. There may have been homologous recombination, however.

Our finding that global regulators are gained and lost more slowly than other regulators complements a report that global regulators, as defined by their weak DNA binding specificity, undergo slower sequence evolution than other regulators (Rajewsky et al., 2002). However, the previous report used bidirectional best BLAST hits to identify orthologous TFs, which can give misleading results (M.N.P. et al., submitted). To confirm that the sequence of global regulators evolves slowly, we examined 40 evolutionary orthologs of characterized TFs between *E. coli* and *S. oneidensis*. These orthologs were identified by an automated analysis of phylogenetic trees (Dehal and Boore, 2006) and were confirmed by inspection. We found a clear correlation between conservation (defined as the BLAST bit score divided by the self score for the *E. coli* gene) and the number of genes that the TF is reported to regulate in RegulonDB (Spearman $rho = 0.48$, $P < 0.002$, $n = 40$). Thus, global regulators do evolve more slowly than other regulators, both in terms of gene gain and gene loss and in their amino acid sequence.

Co-transfer of neighbor regulators with regulated genes

In contrast to global regulators, most neighbor regulators were acquired by horizontal transfer. Neighbor regulators were also significantly more likely than other non-global regulators to be HGT ($P < 0.04$, Fisher exact test). To see if these neighbor regulators were acquired together with genes that they regulate, we asked whether the TF and regulated gene(s) had xenologs that were near each other. (Xenologs are homologs that are related to each other by HGT rather than by vertical descent.) We found that 23 of the 37 neighbor regulators that we inspected have been acquired by co-transfer with one or more of their regulated genes (e.g., xapR with xapA in Figure 3). In contrast, a previous analysis found that bacterial TFs do not usually co-evolve with their regulated genes (Lozada-Chavez et al., 2006). The previous analysis relied on bidirectional best BLAST hits, and for TFs, these hits are often spurious (M.N.P. et al., submitted).

It has also been proposed that repressors are more likely than activators to co-evolve with their regulated genes (Hershberg and Margalit, 2006). However, we found that activators, repressors, and dual regulators were equally likely to be transferred together with their regulated genes (Supplementary Note 1). The discrepancy might arise because we looked for co-transfer events, while the previous work looked for gene loss events. In other words, the regulators are co-evolving with their genes by HGT, regardless of the sign of the regulation, but activators are more likely to be lost, perhaps as the first step towards the loss of the entire pathway (Hershberg and Margalit, 2006). Indeed, both of the the regulators whose loss is discussed in detail in the previous work have undergone co-transfer with regulated genes (flhDC with fliA and fliD, and malT with malS; see Supplementary Note 1). Overall, HGT seems to be associated with neighbor regulation, and a majority of neighbor regulators have been acquired together with their regulated genes.

Most uncharacterized regulators are neighbor regulators

We considered that co-transfer might be used to predict the function of uncharacterized regulators. To see if such predictions would be reliable, we looked for co-transfer events among the 40 non-neighbor-regulators (including global regulators) that we examined. We also looked for co-transfer events involving TFs that are known (Salgado et al., 2006) or predicted (Price et al., 2005b) to be in operons. We found 10 additional co-transfer events, and in 7 of these cases the co-transferred genes are regulated by the TF. (In most of these cases, the TF was not classified as a neighbor regulator because it was co-transcribed with the regulated genes.) The three exceptions were: fecR has been transferred together with its sensor fecI, alpA has been transferred together with yfjI as part of prophage CP4-57 (Trempy et al., 2004), and flhDC has transferred together with motAB, which is also involved in chemotaxis. Overall, co-transfer was not a 100% reliable indicator of regulation, but we found few exceptions relative to the large number of co-transfer events that did indicate regulation (3 vs. 30), and in all cases, the co-transferred genes did have related functions.

When we analyzed, by hand, the evolutionary history of a random sample of 20 putative regulators

that have not yet been characterized, we found that most of them were acquired by HGT (17/20, Figure 6). Almost half of them (9/20) were co-HGT with adjacent genes. This proportion is similar to the proportion of neighbor regulators that are co-HGT (23/37). (The proportions are not significantly different, $P > 0.2$, Fisher exact test.) Hence, we predict that most of the as-yet-uncharacterized regulators in *E. coli* are neighbor regulators. We also predict that most of the uncharacterized regulators control the expression of just one or two operons, as is seen for the characterized neighbor regulators (Hershberg et al., 2005).

We tried to identify co-transfer automatically by searching for conserved proximity in distant organisms, but without much success. We used bidirectional best hits to identify potential orthologs in those organisms, and although these best hits are often false positives, we hypothesized that testing for conserved proximity would eliminate the false positives. Unfortunately, this automated approach did not identify most of the co-transferred TFs that we identified manually (data not shown). Many of the HGT events are between *E. coli* and related bacteria (discussed below), and detailed phylogenetic analysis is required to uncover these HGT events. Conserved proximity has also been used in combination with orthology groups (COGs, Tatusov et al. (2001)) to identify regulatory relationships (Korbel et al., 2004). That study found many successful predictions but also had a high rate of false positives because of the difficulty of automatically placing TFs into orthology groups (Korbel et al., 2004). Thus, automating the identification of co-transfer is beyond the scope of this paper.

Repeated HGT of Regulators Between Related Bacteria

While examining the neighbor regulators, we sometimes found that close homologs of these regulators had sporadic distributions in *E. coli* and its relatives (e.g., xapR in Figure 3). We classified as "repeated HGT" those genes whose sporadic distributions implied two or more HGT events within the γ-Proteobacteria. (As previously, we inferred an HGT event when three or more independent deletion events would otherwise be required to explain the distribution across species of a clade in the gene tree.) By this restrictive definition, we found repeated HGT between relatives for 16 of the 37 neighbor regulators, which indicates both a strong preference for gene transfer within γ-Proteobacteria and high rates of gene gain for this class of genes.

Previous studies have disagreed as to whether HGT of regulatory genes is relatively common (Nakamura et al., 2004) or relatively rare (Beiko et al., 2005). The study that found that HGT of regulatory genes was rare relied on clusters that contained only one gene per genome to define gene families (Beiko et al., 2005). Such clusters might be difficult to identify for large families such as TFs. Although we do not compare the rate of HGT for regulators to the rate of HGT for other types of genes, we find high rates of HGT for regulators, with the exception of a few global regulators (Figure 6).

Previous studies have also disagreed as to whether HGT within the γ-Proteobacteria is prevalent

(Beiko et al., 2005; Homma et al., 2006) or not (Lerat et al., 2005; Kunin et al., 2005). To confirm that HGT between related bacteria is common, we used an automated procedure, based on the presence and absence of close homologs of a gene, to identify potential HGT events (see Methods). We then asked whether the closest xenologs of these HGT genes were from related bacteria. We found that these closest xenologs were far more likely to be from related bacteria than expected by chance ($P < 10^{-15}$, binomial test; Supplementary Figure 1). Because identifying HGT between related genomes requires large numbers of genome sequences, so that the absence of the gene from intermediate genomes can be confirmed (e.g., Figure 3), too few genomes may have been available for previous studies to see this trend. For example, we analyzed 87 γ-Proteobacterial genomes, while Lerat et al. (2005) analyzed only 13 γ-Proteobacteria.

Evolutionary Histories of Regulatory Interactions

Little of Gene Regulation Arises by Duplication

As discussed above, most of the TFs that we analyzed seem to have arisen by HGT events rather than by duplications within the *E. coli* lineage. If we extrapolate from the TFs tabulated in Figure 6, and correct for the uneven sampling of different types of regulators, then 34 $\pm$ 8 of the 256 regulators in *E. coli* arose by lineage-specific duplications, and 161 $\pm$ 9 regulators were acquired by HGT. (We estimated these standard errors by simulating data according to the observed frequencies within each type of regulator, i.e., parametric bootstrap.) Thus, although bacterial TFs form large families that often have many representatives within a single genome, these representatives are largely xenologs that arose by horizontal gene transfer, rather than being evolutionary paralogs that arose by duplication within the *E. coli* lineage.

When we examined the few TFs that did arise by lineage-specific duplication, we found that many of them do not share regulation with their paralogs. We must exclude uncharacterized TFs, and we also excluded auto-regulation, which is reported for over half of the characterized TFs in RegulonDB and which need not be conserved from the common ancestor (see below). Out of 13 lineage-specific duplications, 6 TFs share regulated genes with their paralogs. Combining these results together, we hypothesized that little of gene regulation arises by duplication.

In contrast, an analysis by Teichmann and Babu (2004) found that "more than two-thirds of *E. coli* ... transcription factors have at least one interaction in common with their duplicates." More broadly, they report that "more than one-third of known regulatory interactions [in *E. coli*] were inherited from the ancestral transcription factor or target gene after duplication." However, they identified distant homologs within the *E. coli* by analyzing structural domains. Most of these structural paralogs diverged so long ago that the homology cannot be identified by protein BLAST (data not shown). Because gene regulation in bacteria evolves rapidly (Lozada-Chavez et al. (2006); Gelfand (2006); M.N.P. et al., submitted), we suspected that these paralogs diverged before the current

regulation of these genes evolved. If this is correct, then these regulatory similarities between paralogs were not inherited from a common ancestor, and might instead be due to convergent evolution.

To see if the homologs identified by Teichmann and Babu (2004) diverged before their current regulation evolved, we compared the evolutionary ages of the duplication events and of the gene regulation. We estimated the evolutionary age of the duplication event by examining gene trees. To put an upper bound on the evolutionary age of the gene regulation, we reasoned that the regulatory relationship between a TF and a gene cannot predate the coexistence of those genes in the same genome. For example, the response regulators arcA and dcuR (which is also known as yjdG) were identified as homologs by Teichmann and Babu (2004), and they both regulate dctA (Davies et al., 1999). As shown in Figure 7, dcuR and dctA are present in other Enterobacteria but are absent from more distant γ-Proteobacteria such as *Pasteurella*, *Vibrio*, and *Shewanella*. Thus, these genes were acquired long after the divergence of dcuR and arcA. This also implies that the regulation involved recently, unless all genes were present on both sides of the transfer event. However, dcuR seems to have been acquired from Firmicutes, while arcA is not present in Firmicutes. ArcA's closest homolog in the best-studied Firmicute, *B. subtilis*, is yycF, which seems to be required for viability and may regulate cell wall homeostasis (Fabret and Hoch, 1998; Szurmant et al., 2005), while arcA regulates energy metabolism at low levels of oxygen (Alexeeva et al., 2003). Furthermore, arcA has an ancient paralog torR within Proteobacteria (data not shown), and torR and arcA have different functions, which suggests that arcA's function evolved more recently and that arcA is not present in Firmicutes. Thus, arcA is not present in Firmicutes, and the joint regulation of dctA by arcA and dcuR must have evolved after the transfer of dcuR into the *E. coli* lineage, and long after the divergence of arcA from dcuR.

We repeated this analysis for 30 randomly selected examples of shared regulation between homologous genes from Teichmann and Babu (2004) (see Supplementary Note 2). In most cases, we found that one of the genes had been acquired by HGT relatively recently, and from bacteria that do not appear to contain orthologs of the other genes, so that the regulation presumably evolved after the horizontal transfer event. We also found inconsistent operon structure that seemed to be evidence against evolution by duplication. For example, the paralogous genes tdcE and pflB are both regulated by CRP and IHF. Because tdcE and pflB are in operons, and because the first gene in the two operons are not homologous (tdcA and focA), the regulation of the two operons probably arose independently. Alternatively, the other genes in the operon could have inserted between the promoter and the genes after the duplication, but this seems unlikely; furthermore, changes in operon structure are often accompanied by changes in gene regulation (Price et al., 2006). We confirmed only one of the 30 interactions as evolving by duplication. Thus, most of the regulatory similarities between distant homologs are not inherited from a common ancestor. The pattern that Teichmann and Babu (2004) identified might instead reflect convergent evolution.

To see if closer homologs have a tendency towards shared regulation, we identified homologs within the *E. coli* genome by protein BLAST. We required the score from BLAST to be at least 30% of the self-score for each gene individually. Because this threshold is effective at distinguishing orthologs

within the γ-Proteobacteria from other homologs (Lerat et al., 2003), this threshold should select for paralogs within the γ-Proteobacteria. Of the 14,993 homologous pairs of proteins in *E. coli* K12, this rule selected 1,560 pairs. Given these "close paralogs," and the regulatory interactions between genes and TFs from RegulonDB, we looked for three types of shared regulation between paralogs, as in (Teichmann and Babu, 2004). We identified paralogous TFs that regulated the same gene (e.g., arcA and dcuR regulate dctA, above); paralogous genes regulated by the same TF (e.g., CRP regulates araE and galP); and paralogous TFs that regulate paralogous genes (e.g., cpxR regulates ompC and ompR regulates ompF). As above, we excluded auto-regulation from consideration. A detailed examination of the interactions is given in Supplementary Note 2.

Across all three types of shared regulation, we found that 14% of the regulatory interactions in RegulonDB were shared between paralogs (Table 1). After removing regulation that is more recent than the duplication event and removing shared regulation that has inconsistent operon structure, however, it appears that only 5-8% of the interactions actually evolved by duplication. (The uncertain 3% represent interactions where the relative age of the duplication and of the regulation was unclear, and operon structure could not be used to clarify.) The other 6-9% of interactions arose by convergent evolution between paralogs. One mechanism of convergent evolution was apparent: we found four cases in which an operon was clearly acquired after the duplication of the TFs, and yet the operon was regulated by paralogous TFs that bound to the same sites. Thus, if paralogous TFs maintain overlapping DNA binding specificities, a single site can evolve to bind both TFs.

To see if the amount of shared regulation between close paralogs was greater than would be expected by chance, we randomly shuffled the regulatory network 1,000 times (see Methods). All 1,000 shuffled networks had fewer cases of regulatory similarity between paralogs than were found in the true network. When we considered each type of sharing separately, we found the same result. In particular, the duplication of both TFs and their regulated genes was more common than chance, while a previous report found it to be less common than chance (Teichmann and Babu, 2004). To see if convergent evolution was more common than expected by chance, we compared the regulatory similarities in the shuffled networks to the number of convergent similarities. We found that each type of convergent evolution occured more often in the real network than in any of the shuffled networks. Thus, convergent evolution seems to be a significant factor in the evolution of gene regulation.

We also considered auto-regulation separately. A recent report found a weak but statistically significant similarity in auto-regulation within families of TFs (Lagomarsino et al., 2007). However, among the close paralogs, we did not find any similarity between paralogs in their tendency to auto-regulate. More precisely, we considered pairs of close paralogs of TFs, and we asked whether auto-regulation was correlated for these pairs. We did not find an effect (odds ratio 1.15, $P > 0.5$, Fisher exact test, 66 pairs). Again, the pattern that was identified in the previous work that considered more distant paralogs might result from convergent evolution.

Overall, we found that only 5-8% of regulatory interactions arose by duplication within the *E. coli* lineage. Another 6-9% of regulatory interactions reflect independent (convergent) evolution of

similar regulation for homologous genes. Thus, convergent evolution probably accounts for more of the regulatory interactions than does evolution by duplication. One caveat in our analysis is that these proportions can be expected to rise as more knowledge of the *E. coli* regulatory network becomes available. Missing information from either of two paralogs will cause any duplication of regulation to be missed, so the amount of duplicate regulation that can be identified grows more rapidly than the size of the network. However, because only 13% of the TFs evolved by duplication within the *E. coli* lineage, and because the majority of the regulatory similarities between paralogs reflect convergent evolution, we can still conclude that little of gene regulation has evolved by duplication.

Complex regulation of acquired genes

Although most transcription factors have been acquired by HGT, we also found that most of the global regulators are more ancient. Because the 20 global regulators account for about two-thirds of the regulatory interactions in RegulonDB, we wondered how these global regulators relate to the bulk of *E. coli* genes, which have been acquired by HGT.

In particular, because many of the genes in *E. coli* were acquired by horizontal gene transfer relatively recently, we hypothesized that these genes would have less time to evolve complex regulation. However, when we examined the regulation of HGT genes that were identified by the automated presence/absence approach, we found that HGT genes are significantly *more* likely than native genes to be under complex regulation (Figure 8). We also compared HGT genes to conserved γ-Proteobacterial genes that are reported not to undergo HGT (Lerat et al., 2003), and we again found that the HGT genes had, on average, more complex regulation (data not shown).

When we examined the HGT genes with complex regulation, we found that many of them are regulated by both a neighbor regulator and a global regulator. Indeed, of the genes with characterized regulation in RegulonDB, CRP regulates 48% of the HGT genes but only 23% of the other genes ($P < 10^{-15}$, Fisher exact test). This presumably occurs because CRP regulates carbon source choice and because many of the HGT genes encode the catabolism of specific carbon sources. More generally, we speculate that HGT genes are particularly likely to be niche-specific and hence to require complex regulation. In any case, these results suggest that the evolution of regulation is driven by selection and that it evolves more rapidly than the timescales considered here.

Regulation of acquired genes – evolving new sites vs. acquiring genes with regulatory signals

Given that most of the global regulators are highly conserved within γ-Proteobacteria, and that genes are preferentially transferred within the γ-Proteobacteria, we wondered whether genes would

conserve their regulation across HGT events. To see if genes are acquired together with regulatory signals, we first considered neighbor-regulated genes that have undergone co-HGT with their regulators within the γ-Proteobacteria. In these cases (16 of the 37 neighbor regulators that we examined), it is very likely that the regulation of the gene by the adjacent TF predates the horizontal transfer event. For five of these 16 operons, there is another known regulator for the operons, and in all five cases that regulator is CRP. CRP is conserved in both sequence and DNA-binding specificity across the γ-Proteobacteria: for example, the protein Clp from the distant γ-Proteobacterium *Xanthomonas campestris* is 45% identical to *E. coli* CRP, has a similar DNA-binding specificity, and complements a CRP knockout when cloned into *E. coli* (de Crecy-Lagard et al., 1990; Dong and Ebright, 1992). So, we used a position-specific weight matrix derived from known CRP binding sites in *E. coli* to predict binding sites for CRP upstream of these operons and upstream of their xenologs in other γ-Proteobacteria (see Methods). We found likely binding sites upstream of xenologs for three of the five operons (Table 2). We did not find CRP sites upstream of *E. coli* melAB or its xenologs, perhaps because CRP does not bind this promoter in the absence of melR (Belyaeva et al., 2000). Finally, dsdXA has a conserved CRP binding site in Enterobacteria but the xenolog from *Photobacterium profundus* does not. Overall, this analysis suggested that complex regulation, which in these cases involved both a neighbor regulator and CRP, can be maintained across HGT events.

We then examined the CRP regulon more broadly. As discussed previously, CRP regulates a larger proportion of HGT genes than of native genes. Although CRP has evolutionary orthologs only within β,γ-Proteobacteria (data not shown), most of these HGT genes (81%) have their best hits to more distantly related bacteria. We examined a random sample of 20 of these genes that were putatively acquired from distant bacteria by hand, and we confirmed that most of them (18/20) were acquired from distantly related bacteria. Many of these genes (12/20) have a sporadic distribution of homologs in intermediate related bacteria such as *Vibrio* species, which suggests that there might be a more recent HGT event as well. In this case, we wondered whether the regulation occurred before or after this intermediate HGT event. When we searched for CRP sites upstream of the first gene in the operon in these intermediate species, we found likely regulatory sites for 4 out of 12 genes. Thus, in most cases, these genes have evolved regulatory sites for CRP after their transfer into the *E. coli* lineage. Given that the CRP regulon is the largest in *E. coli*, it is striking that most of this regulation has evolved relatively recently.

We also asked whether other global regulators have binding sites that have been conserved across HGT events within the γ-Proteobacteria. We considered *E.coli* genes that were acquired from other γ-Proteobacteria (according to our automated presence/absence analysis), that are regulated by global regulators, that are the first gene of their operon, and that have upstream matches to weight matrices from DPInteract (Robison et al., 1998). We found 20 genes that matched these criteria, and in just 6 cases, the closest xenolog also has a potential site for the regulator. Because we used a weak threshold to identify sites (6.0 bits), this could be an overestimate. This analysis confirmed that many of the binding sites for global regulators have evolved relatively recently.

Finally, according to our automated analysis, 57% of the HGT genes in *E. coli* were acquired from outside the β, γ-Proteobacteria. Because most *E. coli* transcription factors do not have orthologs in

such distantly related bacteria ((Lozada-Chavez et al., 2006); M.N.P. et al., submitted), most of this regulation probably evolved after the transfer event. Overall, we found a few cases where complex regulation has been conserved across HGT events, but most of the regulation of these HGT genes in *E. coli* seems to have evolved after the genes were acquired.

Discussion

We have shown that the transcription factors of *E. coli* evolved primarily by horizontal gene transfer rather than by duplications within the *E. coli* lineage. Lineage-specific duplication accounts for a small minority of TFs ($< 13\%$) and for an even smaller proportion of regulatory relationships ($5-8\%$). In contrast, most of the TFs (63%) have been acquired by HGT after the divergence of the *E. coli* lineage from *Shewanella* species. These results support the model of "allopatric gene divergence" wherein a TFs' function diverges after HGT moves the TF into a new genome with new selective pressures, and, once the TF's function diverges, it is reacquired (Gogarten et al., 2002). Allopatric divergence avoids the complications of selection for both copies of the gene that arise when two new paralogs are in the same genome. One might imagine that, once reunited in the same genome, there would be crosstalk or conflict between these regulators, but this isn't generally the case. Indeed, even for TFs that underwent duplication within the *E. coli* lineage, only about half of them share binding sites with their paralogs. DNA binding specificity may evolve rapidly: many TFs are neighbor regulators that bind just one or two sites in the genome, so that their DNA binding specificity should not be highly constrained by selection. Paralogous TFs usually respond to different signals as well, but we have not addressed that here.

We found that TFs are often acquired together with their regulated genes, which confirms a suggestion (Hershberg et al., 2005) that neighbor regulation is maintained by HGT. Thus, neighbor regulators can be viewed as being "selfish regulons," as an analog to the selfish operon theory (Lawrence and Roth, 1996; Lawrence, 1999). More precisely, we imagine that the genes themselves and the regulatory relationship between them benefit the host, but the proximity itself may not be of benefit to the host, and is selected for by HGT. It remains unclear how neighbor regulation arises in the first place – we discuss that issue below. We found that many of the putative, as-yet-uncharacterized TFs of *E. coli* have also been transferred together with adjacent genes, so we infer that most of these TFs are also neighbor regulators and that they also regulate just one or two operons (Hershberg et al., 2005).

Although most TFs have been acquired by HGT, most of the global regulators are well conserved within the γ-Proteobacteria. Because these global regulators are responsible for about two-thirds of known regulation, gene regulation could be more conserved than would be implied by the recent origins of the typical TF. However, HGT genes have more complex regulation than native genes, and most of these HGT genes are acquired from distant bacteria in which global regulators are not conserved. Even for genes that were acquired from other γ-Proteobacteria, most of the binding sites

for global regulators that are found in *E. coli* seem not to be conserved across the HGT events. Thus, it appears that on the time scales considered here, regulation evolves rapidly, even though the global regulators evolve slowly.

Non-random Evolution of Gene Regulation

We found two non-random patterns in the evolution of gene regulation. Both of these patterns seem inconsistent with neutral or nearly-neutral theories for the evolution of gene regulation. First, although regulatory similarities between paralogs (either paralogous TFs or paralogous regulated genes) account for 14% of the regulatory interactions, evolutionary analysis shows that these similarities often result from convergent evolution rather than being conserved from the common ancestor. The tendency towards convergent evolution is statistically significant. We propose that paralogs tend to have similar (but distinct) functions, and that selection sometimes causes these paralogs to have similar regulatory interactions. We also found a few cases where a new site has evolved to bind two paralogous TFs that have overlapping DNA binding specificities.

Second, HGT genes tend to be under more complex regulation than native genes, which is surprising. HGT genes have had less time to evolve complex regulation. Also, HGT genes are less highly expressed than native genes ($P < 10^{-15}$; expression levels from (Price et al., 2005a)), which implies weaker selection on their expression levels. We propose that many HGT genes are niche-specific and hence require more complex control, while native genes are (relatively) constitutively expressed. Because our knowledge of gene regulation in *E. coli* is highly incomplete, however, we cannot rule out the possibility that this trend results from some bias in what geneticists choose to study.

Neighbor Regulators as "Selfish," Niche-Specific Regulons

The mechanism by which neighbor regulators form remains unclear. If we examine the closest homologs of neighbor regulators and regulated genes that are *not* near each other, then we usually find that these homologs are not in the same genomes (data not shown), so the proximity doesn't seem to result from deleting intervening genes. We also note that neighbor-regulated genes are more likely than other characterized genes to be in operons instead of transcribed individually ($P < 0.004$, Fisher exact test), so there may be some operons that are evolving "selfishly" along with their regulators, even though the selfish model doesn't seem to apply to operon formation in general (Pal and Hurst, 2004; Price et al., 2005c, 2006; Homma et al., 2006).

We speculate that neighbor regulation might arise because it allows the TF to bind to a single site and regulate both the TF and the regulated operon. To give a concrete scenario, the TF might already regulate the gene, and after a genome rearrangement moves the TF adjacent to the regulated gene, the TF would become auto-regulated. Alternatively, the TF might already regulate its own

transcription (as is common for all TFs), and after an HGT event inserts the regulated operon adjacent to the TF, the preexisting site would regulate the operon's transcription. This would explain why the majority of neighbor regulators are divergent from their regulated genes, and why the divergent orientation is associated with autoregulatory TFs (Korbel et al., 2004; Hershberg et al., 2005). The other neighbor regulators might arise from divergent neighbor regulators by local inversion, as can be seen for xapR (Figure 3).

As an alternative to the selfish theory, neighbor regulation might be selected for because a newly synthesized TF would be closer to its target (Hershberg et al., 2005). However, the time for TFs to find their targets is short regardless of their location: TFs bind to specific sites at rates of around 10^8/M/s, and if the TF has a single site in the genome, then that site's concentration is about 10^{-9} M, so that a newly synthesized TF should find its binding site, anywhere in the genome, in around 10 seconds on average (Halford and Marko, 2004). *In vivo*, the lac repressor finds its target in at most a few minutes (Elf et al., 2007). Thus, we doubt that there is selection for a TF to be encoded near its target site(s).

Regardless of the origin of neighbor regulation, the repeated HGT of neighbor regulators within γ-Proteobacteria suggests that these regulons are niche-specific. Niche-dependent selection for these genes is also consistent with the functional bias of HGT genes (Nakamura et al., 2004) and the metabolic compatibility of acquired genes with the preexisting capabilities of the host (Pál et al., 2005). Conversely, the sporadic distribution of these genes is consistent with the high rate of loss of recently acquired genes (Hao and Golding, 2006). The rapid loss would most likely be neutral, but it could also reflect selection against capabilities that are deleterious if not frequently needed (Wagner, 2003).

Complex Patterns of Horizontal Gene Transfer

We found that HGT of TFs is rampant, and that many genome sequences are required to detect these events, so that the absence of the gene from intermediate groups of bacteria is clear. Because of HGT between related bacteria, simply comparing the gene tree to the species tree (for those species that contain the gene) may not be a sensitive indicator of HGT. We found that HGT of global regulators was rare, but because these regulators are resistant to gene loss, we cannot use gene absence to help us identify HGT. Thus, we could be underestimating the rate of HGT for these genes. As in the case of CRP, these global regulators often have conserved context, so insertion of a xenolog and loss of the original gene seems not to occur. However, homologous recombination could be replacing all or parts these sequences in place, especially given the high conservation of these genes (e.g., the DNA sequence of CRP is 88% identical between *E. coli* and *S. typhimurium* LT2). Indeed, some workers argue that all bacterial genes are subject to frequent HGT events (Doolittle and Bapteste, 2007). In this case, the distinction between HGT and other genes might not be meaningful, but there remains a difference between genes that are frequently gained and lost (i.e., niche-specific neighbor regulators), and genes that have occasionally undergone recombination (i.e.,

global regulators).

Why should HGT between related bacteria be prevalent? In general, the divergence of the genes involved seems to be too great for homologous recombination. Also, because neighbor regulators are often acquired together with a regulated operon, it might not seem necessary for other machinery in the host to be conserved. However, neighbor-regulated operons are also often regulated by global regulators such as CRP, and we did find some cases where CRP binding sites were conserved across transfer events. Even if the operon has only one regulator, differences in the core transcriptional machinery in different hosts might prevent the newly acquired neighbor regulator from functioning, especially for activators. Another possibility is that related bacteria are more likely to have genes that fit into the preexisting metabolic pathways of the new host, which increases the likelihood of HGT (Pál et al., 2005).

Methods

Regulatory Interactions

We obtained regulatory interactions from RegulonDB 5.6 (Salgado et al., 2006). After removing RNA genes and pseudogenes, and the housekeeping sigma factor rpoD, we had 159 TFs, 1,354 regulated genes, and 3,085 regulatory interactions between them. A few of the TFs are heterodimers – these were counted only once. We also examined TF and gene annotations in EcoCyc (Keseler et al., 2005) and known operons in RegulonDB.

Evolutionary histories of TFs

We investigated the evolutionary histories of TFs by comparing the gene tree to the species tree. As a first step, we used fast neighbor-joining trees (Howe et al., 2002) for COGs, PFams, and ad-hoc BLAST families from the MicrobesOnline tree-browser (http://www.microbesonline.org/treebrowseHelp.html), and we compared the gene trees to the MicrobesOnline species tree (the most relevant parts of the species tree are shown in Figure 2, and the construction of the species tree is described below).

Given a gene tree and a species tree, we identified horizontal transfer events using a combination of the gene phylogeny and the pattern of gene presence and gene absence. If a strongly supported clade in the gene tree was present in disparate genomes, so that three or more deletion events would be required to explain the distribution of the subfamily on the species tree, then we assigned an HGT event. Deletions in the highly reduced genomes of the insect endosymbiont group (*Buchnera*, *Wigglesworthia*, and *Blochmannia*) were not considered as evidence for HGT. Given that HGT

seems to be common in bacteria, the threshold of three or more deletion events is conservative. In particular, with higher thresholds, a large number of deletions from ancestral bacteria are required to explain the present distribution of genes, which requires the ancestral bacteria to have had unreasonably large genomes (Mirkin et al., 2003; Kunin and Ouzounis, 2003).

If the gene tree showed paralogs, and the phylogeny of two subgroups was consistent with the species tree, then we assigned a gene duplication event. Histories that did not meet either of these criteria were considered native, even if there were minor discrepancies between the species tree and the gene tree.

Once we had a tentative classification, we confirmed it by checking for close homologs (by BLASTp) that might be absent from the gene family (due to the limitations of gene family assignment) and by building a smaller and more accurate phylogenetic tree for a selected subset of homologs. To build these higher-quality trees, we used MUSCLE (Edgar, 2004) to align the protein-coding sequences, Gblocks to trim the alignments (Castresana, 2000), and both TreePuzzle (Schmidt et al., 2002) and phyml (Guindon and Gascuel, 2003) to build phylogenetic trees.

We also asked whether the putative HGT event affected the *E. coli* lineage. For example, as seen for CRP (Figure 5), the tree suggests a transfer event from *E. coli*'s ancestors to another lineage, but this does not imply that *E. coli*'s ancestors acquired the gene by HGT. These genes were classified as native.

We assume that these genes were transferred from other bacteria into the *E. coli* lineage, rather than *vice versa*, even though it is theoretically possible that these TFs arose in the *E. coli* lineage relatively recently and were then transferred elsewhere. Because most of the TFs belong to large families that are present in many other bacterial lineages, and also because these TFs often have distant paralogs in *E. coli*, a recent origin of these families within the *E. coli* lineage is not plausible.

Species tree

The species tree was computed from maximum likelihood trees of concatenated proteins by using matrix representation of parsimony (Ragan, 1992). The maximum likelihood trees were generated from a lower-quality guide tree by selecting, for each internal node in the guide tree, a small number of descendant genomes and close out-groups (less than 20 genomes total). Given this small group of genomes, we identified COGs (Tatusov et al., 2001) that are present as a single copy in each genome. Because these groups of genomes usually consisted of close relatives, there were typically hundreds of conserved genes. We aligned and trimmed each COG, again using MUSCLE and Gblocks, and concatenated the alignments. Because the resulting alignments were often very large, we removed invariant sites, and if the alignment still contained over 5,000 positions, we took a random sample of sites. We then built a tree with phyml, using 4 categories of evolutionary rates. We converted the trees to a matrix of characters (Ragan, 1992) and used PAUP 4.0b10 (Swofford, 2003) to infer

the most parsimonious tree. Finally, we used PHYLIP (http://evolution.genetics.washington.edu/phylip.html) to infer maximum likelihood branch lengths, with gamma-distributed rates, from a concatenated alignment of 74 highly-conserved proteins.

A fuller description of the species tree construction is given in http://www.microbesonline.org/treebrowseHelp.html#speciestree.) The tree does not contain bootstrap values, but most of the source trees have strong bootstrap support and are congruent with each other (data not shown). The most relevant uncertainties are (1) the placement of *Photorhabdus*, and (2) whether or not *Sodalis* should be grouped with the other insect endosymbionts (*Buchnera*, etc.).

Sampling of regulators

We examined all of the top 20 global regulators, which account for about two-thirds of the regulatory interactions in RegulonDB. For neighbor regulators, we examined those that were described in an earlier compilation of regulatory interactions, ColiNet 1.1 (Shen-Orr et al., 2002), which we used in the initial phase of this project. Although this is not a truly random sample, we do not know of any reason why the more recently characterized regulators would have different evolutionary histories. We examined a random sample of 25 of the other characterized regulators in RegulonDB, which grew to 26 because of a heterodimer. Again, these were primarily regulators that were described in ColiNet.

We identified putative regulators in *E. coli* K12 by searching for gene ontology GO:0003700 ("transcription factor activity") using the MicrobesOnline database. We randomly selected 20 of these to examine, and we verified that they were predicted to contain helix-turn-helix domains (by using InterPro), that they were not annotated as restriction enzymes or DNA modification enzymes, and that they were not already characterized (by using ecocyc).

Automatic identification of HGT genes

To identify HGT automatically, we looked for genes that lack close homologs in consecutive groups of related bacteria (Figure 9). We defined "close" homologs by BLAST scores, and to confirm the putative HGT, we used a quartet test (see Figure 9). This approach contrasts to approaches that rely heavily on the gene tree (Beiko et al., 2005), and is more similar to presence/absence analyses (Lerat et al., 2005). Although the method is conservative, and misses many HGT events (data not shown), it classifies about a quarter of protein-coding genes in *E. coli* K12 as HGT, which yields a sufficiently large sample for analysis.

The quartet test was not conducted if there was no more distant homolog in each of the groups of genomes that were "missing" good hits to the gene, as in these cases we do not have 4 genes to form a quartet out of. If we did have a gene from each group of genomes, we aligned the four genes with

MUSCLE, we removed positions with gaps, and we tested the likelihood of all three topologies with tree-puzzle (Schmidt et al., 2002), using gamma-distributed evolutionary rates.

Shuffled Regulatory Network

For each shuffle, we selected the regulated genes for each TF by sampling without replacement from the complete set of regulated genes, so that the number of interactions for each TF and for each regulated gene was unchanged. We re-sampled parts of the network to avoid duplicate interactions between regulated genes and TFs. This gave networks with the same degree distribution as the original network, both for TFs and for regulated genes.

Predicting binding sites for global regulators

We obtained characterized CRP binding sites in *E. coli* from DPInteract (Robison et al., 1998). We aligned these sites with MEME (Bailey and Elkan, 1995), converted the alignment to a weight matrix with palindromic symmetry, and used patser (Hertz and Stormo, 1999) to search for sites. We searched from -200 to +100 relative to each gene's start codon, and we considered only potential sites with a score of 6.0 bits or higher. This cutoff is quite weak, which increases our confidence that the gene is not regulated by CRP if no site is found, but it raises the question of whether the sites that are conserved across HGT events (Table 2) might have been found by chance. Although 13% of randomly selected upstream regions for xenologs of *E. coli* genes had a hit at 6.0 bits or above, the sites in Table 2 are much stronger: yiaK and gntK have over 10 bits, which occurs in less than 1% of upstream regions, and araB has two nearby sites, which suggests cooperative binding and is also unlikely to occur by chance.

Analyses for other global regulators that have weight matrices in DPInteract were conducted similarly, but without forcing the weight matrix to be palindromic. Some of the sigma factors have multiple models, in which case we used the best score for any model. The weight matrices for lrp and fis were not used because they have poor specificity (Robison et al., 1998).

Acknowledgments

This work was supported by a grant from the US Department of Energy Genomics:GTL program (DE-AC02-05CH11231). A.P.A. would also like to acknowledge the support of the Howard Hughes Medical Institute.

References

Alexeeva, S., Hellingwerf, K. J., and de Mattos, M. J. T. (2003). Requirement of ArcA for redox regulation in Escherichia coli under microaerobic but not anaerobic or aerobic conditions. *J Bacteriol.*, 185:204–9.

Babu, M. M. and Teichmann, S. A. (2003). Evolution of transcription factors and the gene regulatory network in Escherichia coli. *Nucleic Acids Res.*, 31:1234–1244.

Bailey, T. L. and Elkan, C. (1995). Unsupervised learning of multiple motifs in biopolymers using expectation maximization. *Machine Learning*, 21:51–80.

Beiko, R. G., Harlow, T. J., and Ragan, M. A. (2005). Highways of gene sharing in prokaryotes. *Proc. Natl. Acad. Sci. USA*, 102:14332–7.

Belyaeva, T. A., Wade, J. T., Webster, C. L., Howard, V. J., Thomas, M. S., Hyde, E. I., and Busby, S. J. (2000). Transcription activation at the Escherichia coli melAB promoter: the role of MelR and the cyclic AMP receptor protein. *Mol Microbiol.*, 36:211–22.

Castresana, J. (2000). Selection of conserved blocks from multiple alignments for their use in phylogenetic analysis. *Mol Biol Evol.*, 17:540–52.

Davies, S. J., Golby, P., Omrani, D., Broad, S. A., Harrington, V. L., Guest, J. R., Kelly, D. J., and Andrews, S. C. (1999). Inactivation and regulation of the aerobic C(4)-dicarboxylate transport (dctA) gene of Escherichia coli. *J. Bacteriol.*, 181:5624–35.

de Crecy-Lagard, V., Glaser, P., Lejeune, P., Sismeiro, O., Barber, C. E., Daniels, M. J., and Danchin, A. (1990). A Xanthomonas campestris pv. campestris protein similar to catabolite activation factor is involved in regulation of phytopathogenicity. *J Bacteriol.*, 172:5877–83.

Dehal, P. S. and Boore, J. L. (2006). A phylogenomic gene cluster resource: the Phylogenetically Inferred Groups (PhIGs) database. *BMC Bioinformatics*, 7:201.

Dong, Q. and Ebright, R. H. (1992). DNA binding specificity and sequence of Xanthomonas campestris catabolite gene activator protein-like protein. *J Bacteriol.*, 174:5457–61.

Doolittle, W. F. and Bapteste, E. (2007). Pattern pluralism and the Tree of Life hypothesis. *Proc. Natl. Acad. Sci. USA*, 104:2043–9.

Edgar, R. C. (2004). MUSCLE: multiple sequence alignment with high accuracy and high throughput. *Nucleic Acids Res.*, 32:1792–1797.

Elf, J., Li, G.-W., and Xie, X. S. (2007). Probing transcription factor dynamics at the single-molecule level in a living cell. *Science*, 316:1191–1194.

Fabret, C. and Hoch, J. A. (1998). A two-component signal transduction system essential for growth of Bacillus subtilis: implications for anti-infective therapy. *J Bacteriol.*, 180:6375–83.

Gelfand, M. S. (2006). Evolution of transcriptional regulatory networks in microbial genomes. *Curr Opin Struct Biol.*, 16:420–9.

Gogarten, J. P., Doolittle, W. F., and Lawrence, J. G. (2002). Prokaryotic evolution in light of gene transfer. *Mol Bio Evol.*, 19:2226–2238.

Guindon, S. and Gascuel, O. (2003). A simple, fast, and accurate algorithm to estimate large phylogenies by maximum likelihood. *Syst Biol.*, 52:696–704.

Halford, S. E. and Marko, J. F. (2004). How do site-specific DNA-binding proteins find their targets? *Nucleic Acids Res.*, 32:3040–3052.

Hao, W. and Golding, G. B. (2006). The fate of laterally transferred genes: Life in the fast lane to adaptation or death. *Genome Res.*, 16:636–643.

Hershberg, R. and Margalit, H. (2006). Co-evolution of transcription factors and their targets depends on mode of regulation. *Genome Biol.*, 7:R62.

Hershberg, R., Yeger-Lotem, E., and Margalit, H. (2005). Chromosome organization is shaped by the transcription regulatory network. *Trends Genet.*, 21:138–142.

Hertz, G. Z. and Stormo, G. D. (1999). Identifying DNA and protein patterns with statistically significant alignments of multiple sequences. *Bioinformatics*, 15:563–77.

Homma, K., Fukuchi, S., Nakamura, Y., Gojobori, T., and Nishikawa, K. (2006). Gene cluster analysis method identifies horizontally transferred genes with high reliability and indicates that they provide the main mechanism of operon gain in 8 species of γ-Proteobacteria. *Mol. Biol. Evol.*, 24:805–813.

Howe, K., Bateman, A., and Durbin, R. (2002). Quicktree: building huge neighbour-joining trees of protein sequences. *Bioinformatics*, 18:1546–7.

Janga, S. C. and Moreno-Hagelsieb, G. (2004). Conservation of adjacency as evidence of paralogous operons. *Nucleic Acids Res.*, 32:5392–7.

Keseler, I. M., Collado-Vides, J., Gama-Castro, S., Ingraham, J., Paley, S., Paulsen, I. T., Peralta-Gil, M., and Karp, P. D. (2005). EcoCyc: a comprehensive database resource for Escherichia coli. *Nucleic Acids Res.*, 33:D334–7.

Korbel, J. O., Jensen, L. J., von Mering, C., and Bork, P. (2004). Analysis of genomic context: prediction of functional associations from conserved bidirectionally transcribed gene pairs. *Nat. Biotechnol.*, 22:911–7.

Kunin, V., Goldovsky, L., Darzentas, N., and Ouzounis, C. A. (2005). The net of life: reconstructing the microbial phylogenetic network. *Genome Res.*, 15:954–9.

Kunin, V. and Ouzounis, C. A. (2003). The balance of driving forces during genome evolution in prokaryotes. *Genome Res.*, 13:1589–1594.

Lagomarsino, M. C., Jona, P., Bassetti, B., and Isambert, H. (2007). Hierarchy and feedback in the evolution of the Escherichia coli transcription network. *Proc. Natl. Acad. Sci. USA*, 104:5516–5520.

Lawrence, J. G. (1999). Selfish operons: the evolutionary impact of gene clustering in prokaryotes and eukaryotes. *Curr. Opin. Genet. Dev.*, 9:642–8.

Lawrence, J. G. and Roth, J. R. (1996). Selfish operons: horizontal transfer may drive the evolution of gene clusters. *Genetics*, 143:1843–60.

Lerat, E., Daubin, V., and Moran, N. A. (2003). From gene trees to organismal phylogeny in prokaryotes: The case of the gamma-Proteobacteria. *PLoS Biol.*, 1:E19.

Lerat, E., Daubin, V., Ochman, H., and Moran, N. A. (2005). Evolutionary origins of genomic repertoires in bacteria. *PLoS Biol.*, 3:e130.

Lozada-Chavez, I., Janga, S. C., and Collado-Vides, J. (2006). Bacterial regulatory networks are extremely flexible in evolution. *Nucleic Acids Res.*, 34:3434–45.

McCue, L. A., Thompson, W., Carmack, C. S., and Lawrence, C. E. (2002). Factors influencing the identification of transcription factor binding sites by cross-species comparison. *Genome Res.*, 12:1523–32.

McCue, L. A., Thompson, W., Carmack, C. S., Ryan, M. P., Liu, J. S., Derbyshire, V., and Lawrence, C. E. (2001). Phylogenetic footprinting of transcription factor binding sites in proteobacterial genomes. *Nucleic Acids Res.*, 29:774–782.

Mirkin, B. G., Fenner, T. I., Galperin, M. Y., and Koonin, E. V. (2003). Algorithms for computing parsimonious evolutionary scenarios for genome evolution, the last universal common ancestor and dominance of horizontal gene transfer in the evolution of prokaryotes. *BMC Evol Biol.*, 3:2.

Nakamura, Y., Itoh, T., Matsuda, H., and Gojobori, T. (2004). Biased biological functions of horizontally transferred genes in prokaryotic genomes. *Nat Genet.*, 36:760–6.

Otsuka, J., Watanabe, H., and Mori, K. T. (1996). Evolution of transcriptional regulation system through promiscuous coupling of regulatory proteins with operons: Suggestion from protein sequence similarities in Escherichia coli. *J. theor. Biol.*, 178:183–204.

Pal, C. and Hurst, L. D. (2004). Evidence against the selfish operon theory. *Trends Genet.*, 20:232–4.

Price, M. N., Alm, E. J., and Arkin, A. P. (2005a). Interruptions in gene expression drive highly expressed operons to the leading strand of dna replication. *Nucleic Acids Research*, 33:3224–34.

Price, M. N., Arkin, A. P., and Alm, E. J. (2006). The life-cycle of operons. *PLoS Genet.*, 2:e96.

Price, M. N., Huang, K. H., Alm, E. J., and Arkin, A. P. (2005b). A novel method for accurate operon predictions in all sequenced prokaryotes. *Nucleic Acids Res.*, 33:880–92.

Price, M. N., Huang, K. H., Alm, E. J., and Arkin, A. P. (2005c). Operon formation is driven by co-regulation and not by horizontal gene transfer. *Genome Res.*, 15:809–19.

Pál, C., Papp, B., and Lercher, M. J. (2005). Adaptive evolution of bacterial metabolic networks by horizontal gene transfer. *Nat. Genet.*, 37:1372–1375.

Ragan, M. A. (1992). Phylogenetic inference based on matrix representation of trees. *Mol Phylogenet Evol.*, 1:53–8.

Rajewsky, N., Socci, N. D., Zapotocky, M., and Siggia, E. D. (2002). The evolution of DNA regulatory regions for proteo-gamma bacteria by interspecies comparisons. *Genome Res.*, 12:298–308.

Robison, K., McGuire, A. M., and Church, G. M. (1998). A comprehensive library of DNA-binding site matrices for 55 proteins applied to the complete Escherichia coli K-12 genome. *J. Mol. Biol.*, 284:241–54.

Salgado, H., Santos-Zavaleta, A., Gama-Castro, S., Peralta-Gil, M., Penaloza-Spinola, M. I., Martinez-Antonio, A., Karp, P. D., and Collado-Vides, J. (2006). The comprehensive updated regulatory network of Escherichia coli K-12. *BMC Bioinformatics*, 7:5.

Salmon, K. A., Hung, S. P., Steffen, N. R., Krupp, R., Baldi, P., Hatfield, G. W., and Gunsalus, R. P. (2005). Global gene expression profiling in Escherichia coli K12: effects of oxygen availability and ArcA. *J Biol Chem.*, 280:15084–96.

Schmidt, H. A., Strimmer, K., Vingron, M., and von Haeseler, A. (2002). TREE-PUZZLE: maximum likelihood phylogenetic analysis using quartets and parallel computing. *Bioinformatics*, 18:502–504.

Seeger, C., Poulsen, C., and Dandanell, G. (1995). Identification and characterization of genes (xapA, xapB, and xapR) involved in xanthosine catabolism in Escherichia coli. *J Bacteriol.*, 177:5506–16.

Shen-Orr, S. S., Milo, R., Magnan, S., and Alon, U. (2002). Network motifs in the transcription regulation network of Escherichia coli. *Nat Genet.*, 31:64–8.

Swofford, D. L. (2003). *PAUP*. Phylogenetic Analysis Using Parsimony (*and Other Methods). Version 4.* Sinauer Associates, Sunderland, Massachusetts.

Szurmant, H., Nelson, K., Kim, E. J., Perego, M., and Hoch, J. A. (2005). YycH regulates the activity of the essential YycFG two-component system in Bacillus subtilis. *J Bacteriol.*, 187:5419–26.

Tatusov, R. L., Natale, D. A., Garkavtsev, I. V., Tatusova, T. A., Shankavaram, U. T., Rao, B. S., Kiryutin, B., Galperin, M. Y., Fedorova, N. D., and Koonin, E. V. (2001). The COG database: new developments in phylogenetic classification of proteins from complete genomes. *Nucleic Acids Res.*, 29:22–8.

Teichmann, S. A. and Babu, M. M. (2004). Gene regulatory network growth by duplication. *Nature Genet.*, 36:492–496.

Trempy, J. E., Kirby, J. E., and Gottesman, S. (2004). Alp suppression of Lon: dependence on the slpA gene. *J Bacteriol.*, 176:2061–2067.

Wagner, A. (2003). Risk management in biological evolution. *J. Theor. Biol.*, 225:45–57.

Yin, Y. and Fischer, D. (2006). On the origin of microbial ORFans: quantifying the strength of the evidence for viral lateral transfer. *BMC Evol Biol.*, 6:63.

Zhang, A., Rimsky, S., Reaban, M. E., Buc, H., and Belfort, M. (1996). Escherichia coli protein analogs StpA and H-NS: regulatory loops, similar and disparate effects on nucleic acid dynamics. *EMBO J.*, 15:1340–9.

Table 1: Evolution of gene regulation by duplication or by convergent evolution. For each case of shared regulation between paralogs, we examined the evolutionary histories of the duplicated genes to see if the regulation was likely to be conserved from the common ancestor. If yes, then the regulatory similarity evolved by duplication; if not, then the similarity results from convergent evolution. For cases where two paralogous TFs regulate the same operon, we also asked if the TFs bind to the same site. For cases where two paralogous genes are regulated by the same TF, we also asked if the first genes in the operons were homologous, as would be expected for evolution by duplication. We tabulate the results below (see Supplementary Note 3 for the individual interactions). Because some regulatory interactions are shared with paralogs in more than one way, the totals are smaller than the sums over the types.

**Type of Shared Regulation**	**Interactions**	**Percentage**
**All Three Types of Shared Regulation, Combined**	425	14.2%
**Evolved by duplication**	145	**4.8%**
**Unclear**	94	**3.1%**
**Convergent evolution**	186	**6.2%**
Interactions that are not shared with paralogs	2,570	85.8%
All of RegulonDB (with auto-regulation removed)	2,995	100.0%
**Type 1: Paralogous TFs Regulate the Same Genes**	212	7.1%
Evolved by duplication	84	2.8%
Unclear	64	2.1%
Relative ages are unclear, and TFs bind the same site	62	2.1%
Duplication is recent, but TFs bind different sites	2	0.1%
Convergent evolution	64	2.1%
Duplication is recent and TFs bind different sites	26	0.9%
Duplication is recent and TFs bind the same sites	28	0.9%
Duplication is recent, sites not known	10	0.3%
**Type 2: Paralogous Genes Are Regulated by the Same TF**	290	9.7%
Evolved by duplication	76	2.5%
Unclear	26	0.9%
Convergent evolution	188	6.3%
Differences in operon structure	166	5.5%
Operons are consistent, but acquired after duplication	22	0.7%
**Type 3: Paralogous TFs Regulate Paralogous Genes**	54	1.8%
Evolved by duplication (similar ages)	8	0.3%
Convergent evolution	46	1.5%
Complex HGT of regulated genes after TF duplication	16	0.5%
TF duplication precedes that of regulated genes	30	1.0%

Table 2: Binding sites for CRP upstream of *E. coli* operons and their xenologs. These *E. coli* operons are regulated by CRP as well as by an adjacent regulator and have been transferred, together with their neighbor regulators, between the *E. coli* lineage and other γ-Proteobacteria. We used a weight matrix to identify potential binding sites for CRP upstream of these operons and their xenologs. For each site we report its sequence, its score in bits, and its position relative to the start codon of the first gene in the operon. The sites that were used to build the weight matrix have 8.41 $\pm$ 2.66 bits (mean $\pm$ standard deviation). Within each site's sequence, positions that match the consensus nAnTGTGAnnnnnnTCACAnTn are capitalized.

**Operon**	**Organism**	**Position**	**Score**	**Site Sequence**
yiaKLMNOPQRS	*E. coli* K12	-175	9.1	aAgTGTGccgtagtTCACgaTc
yiaKLMNOPQRS	*Haemophilus influenzae* RD KW20	-148	10.3	aAaTagGAtctagaTCACAaaa
araBAD	*E. coli* K12	-131	9.1	ttaTtTGcacggcgTCACAcTt
araBDA?C	*V. parahaemolyticus* RIMD 2210633	-177	6.3	tggaGTtcgatgagagcCggTt
”	”	-137	6.5	cgacaTGAtgacgacgAtcgcc
gntKU	*E. coli* K12	-171	13.1	aAaTtTGAagtagcTCACAcTt
gntK-edd	*V. cholerae*	-131	11.5	gttTGTGttatagcTCACAtTt

Figure 1: Evolutionary history of regulators and regulatory interactions. (A) Most of the transcription factors regulate adjacent genes. These "neighbor regulators" are often transferred between related bacteria, and are often lost, so they seem to be niche-specific. Neighbor-regulated genes are often regulated by other regulators as well, but this regulation is usually not conserved across HGT events. (B) Although regulatory interactions can evolve by duplication, duplicated TFs often diverge in function, so that they do not share regulated genes. Even when paralogous TFs (or paralogous regulated genes) have similar regulatory interactions, this more often results from convergent evolution than from evolution by duplication.

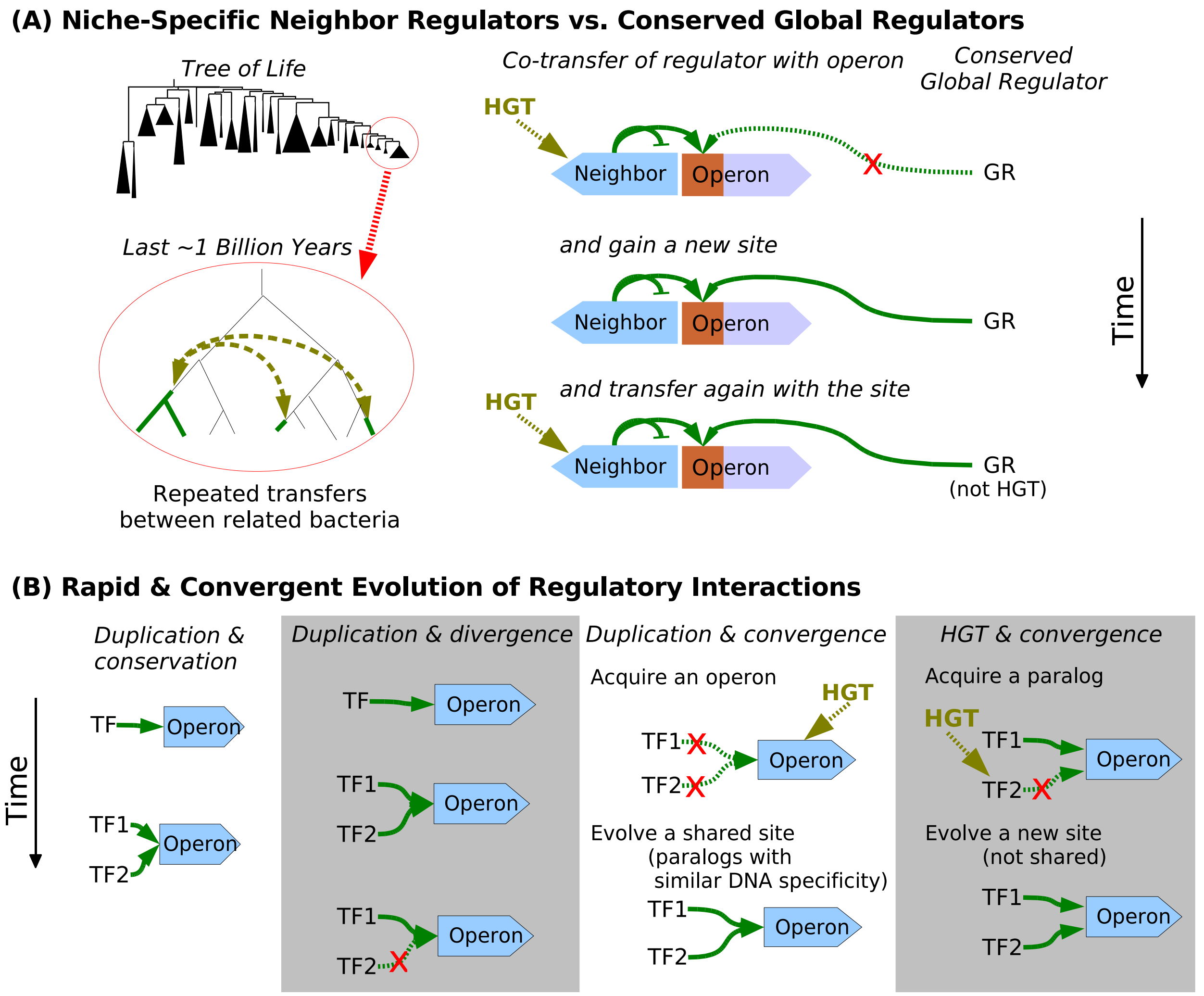

Figure 2: Phylogeny of the γ-Proteobacteria. The phylogeny was derived from concatenated alignments of highly conserved proteins (see Methods). In this study, we focused on evolutionary events after the divergence of *Shewanella* species from *Escherichia coli* K12 (the shaded portion of the tree). The β-Proteobacteria formed a sister group to the γ-Proteobacteria.

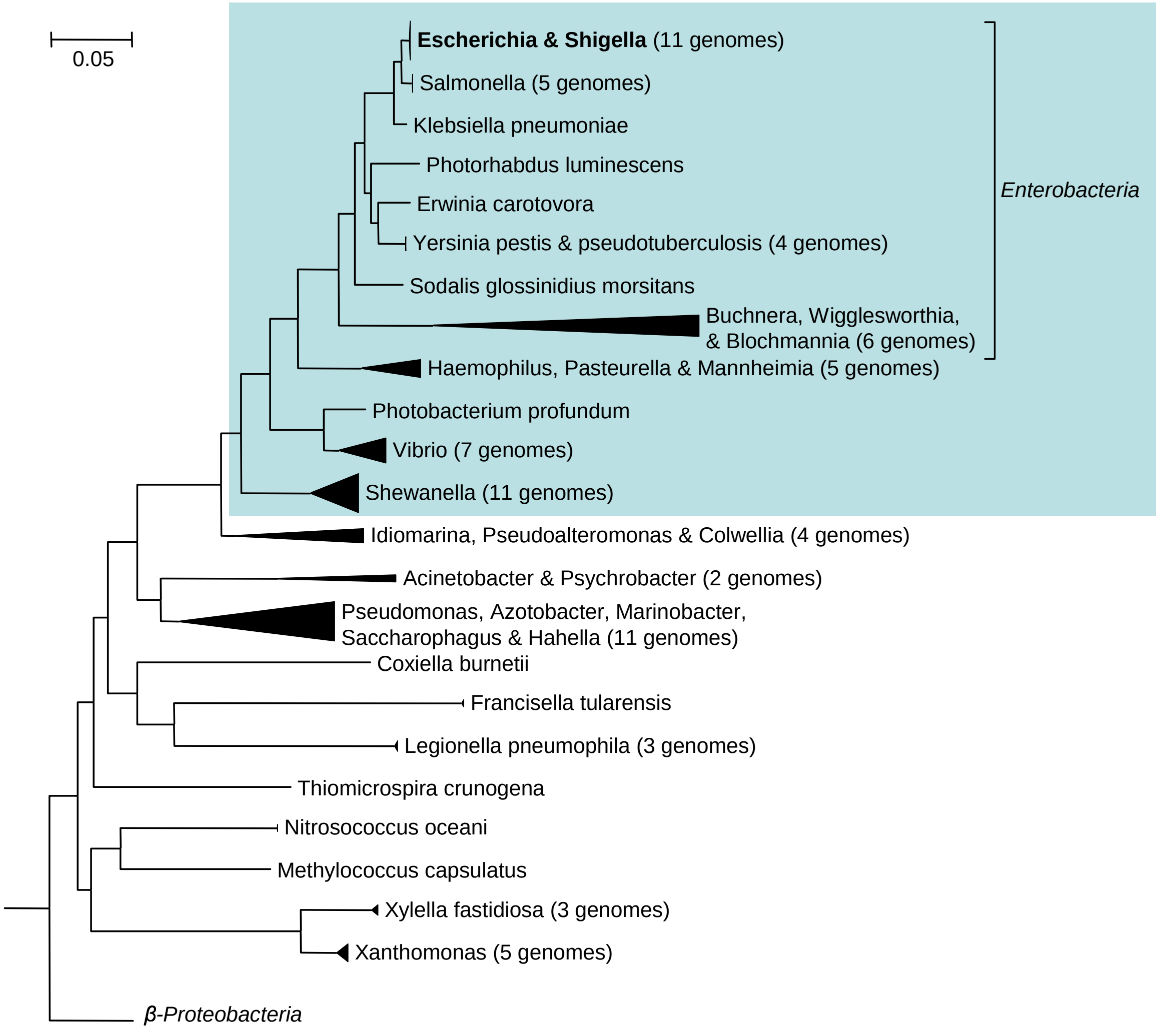

Figure 3: Repeated transfer of xapR together with xapA, which it regulates. In the presence of xanthosine, xapR activates the transcription of the xapAB operon, which allows the transport and catabolism of xanthosine (Seeger et al., 1995). The gene tree shows that xapR forms a well-supported clade (80/100 bootstraps) within a larger family of regulators (COG583). xapR is scattered across the γ-Proteobacteria, within which we identify four acquisition events. For each acquisition, we show the multiple independent gene losses that would otherwise be required to explain the gene's distribution across the species tree. The gene tree also places xapR from *Shewanella baltica* between the sequences from *Vibrio* species, which suggests that it could have been acquired separately by the two groups of *Vibrio*. However, this potential fifth acquisition event is rejected by several criteria: the bootstrap support is low; a small change to the tree's topology (one swap) would render the gene tree congruent with the species tree; and the gene might have been transferred from an ancestor of one of these *Vibrio* species to *S. baltica*. The xapR tree was computed from amino acid sequences using phyml with 100 bootstraps, 4 classes of gamma-distributed rates (with optimized alpha), and an optimized proportion of invariant sites (Guindon and Gascuel, 2003). The gene context shows gene order only (not spacing or scale).

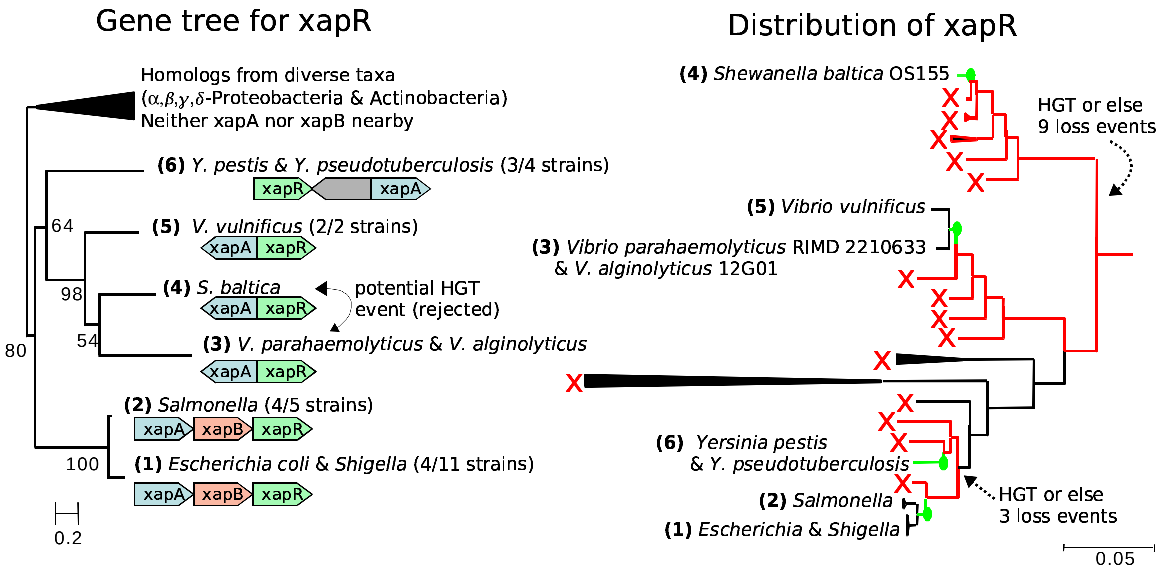

Figure 4: The regulator purR evolved by duplication from the ribose repressor rbsR, which was itself acquired by HGT. As seen in *Ralstonia*, the closest relatives of rbsR and purR from outside the γ-Proteobacteria are associated with genes for ribose utilization and likely function as ribose repressors. Within the Enterobacteria/Vibrionaceae subgroup of the γ-Proteobacteria, both rbsR and purR show largely vertical evolution. The absence of both genes from *Buchnera* and its relatives and from *Sodalis* might suggest additional transfer events, but because *Buchnera* and its relatives have under 700 genes, absence from this clade is not evidence for HGT. *Sodalis* is also a reduced genome, with around 2,600 genes, whereas most Enterobacteria have over 4,000 genes. The purR/rbsR tree was computed from protein sequences with phyml and 100 bootstraps (as in Figure 3).

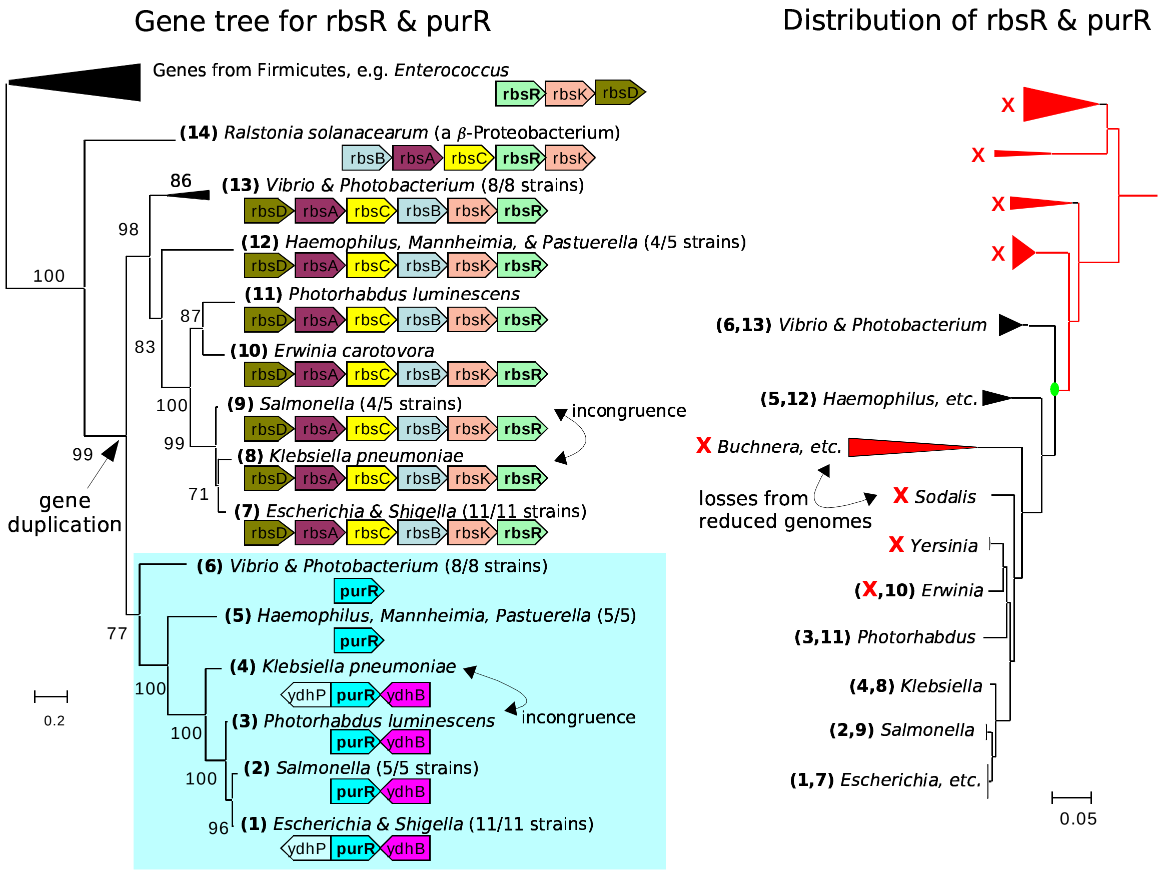

Figure 5: The global regulator CRP has undergone predominantly vertical evolution. CRP has conserved context, and the gene tree is concordant with the species tree except perhaps for the Pasteurellacea and perhaps *Sodalis*. The incongruent placement of *Sodalis* is not supported by a nucleotide sequence tree (data not shown). The deep branching of the Pasteurellacea is strongly supported, and two swaps would be required to make its placement concordant with the species tree. An insertion of CRP into Pasteurellacea is unlikely because of the conserved proximity of the functionally unrelated gene yheT. Instead, the placement probably reflects homologous recombination or long branch attraction. In any case, this does not affect the lineage leading to *E. coli*, and so we classified CRP as native. The CRP tree shown was computed from protein sequences with phyml and 100 bootstraps (as in Figure 3).

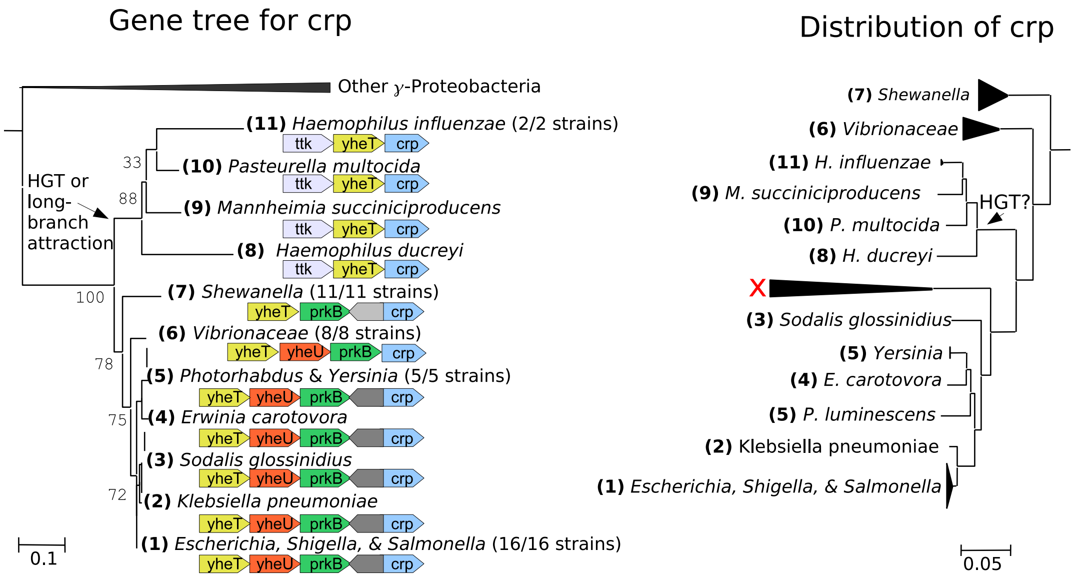

Figure 6: Evolutionary histories of *E. coli* transcription factors. We classified characterized regulators as global regulators, neighbor regulators, or other regulators, and we also analyzed some putative (as-yet-uncharacterized) regulators. We classified these TFs as native since the divergence of *E. coli* from *Shewanella*, as acquired by horizontal transfer after that divergence, as ORFan (indicating HGT from an unknown source), or as duplications within the *E. coli* lineage. For the duplicated TFs, we examined whether they regulate the same genes as their duplicates. For the HGT regulators, we examined whether they were transferred together with adjacent genes and whether they underwent repeated HGT within γ-Proteobacteria.

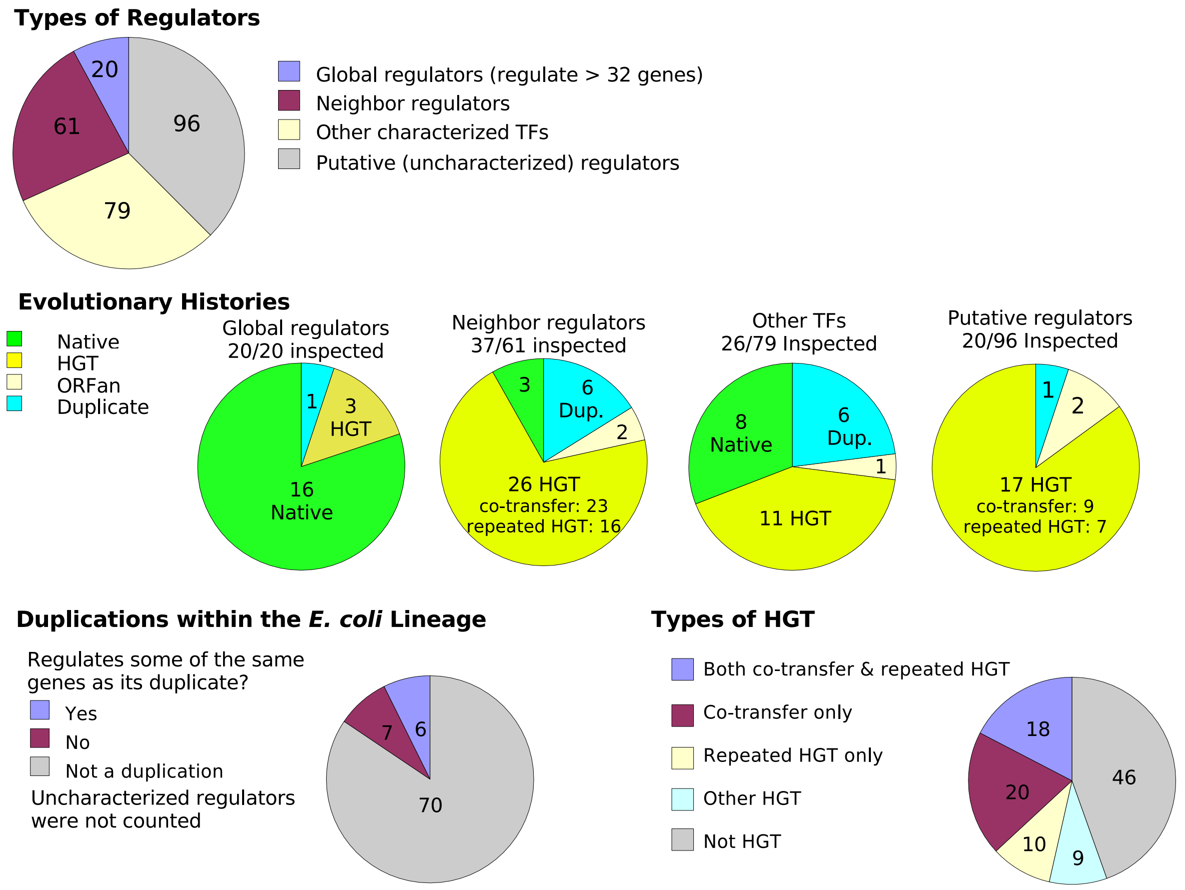

Figure 7: Convergent evolution of regulation of dctA by two distantly related response regulators. From the gene trees (not shown), we identified clades that correspond to dctA, dcuR, and arcA. We show the presence and absence of genes from these clades within the γ-Proteobacteria. The coexistence of dcuR and dctA in the genome is relatively recent, which shows that this regulation evolved long after dcuR diverged from arcA.

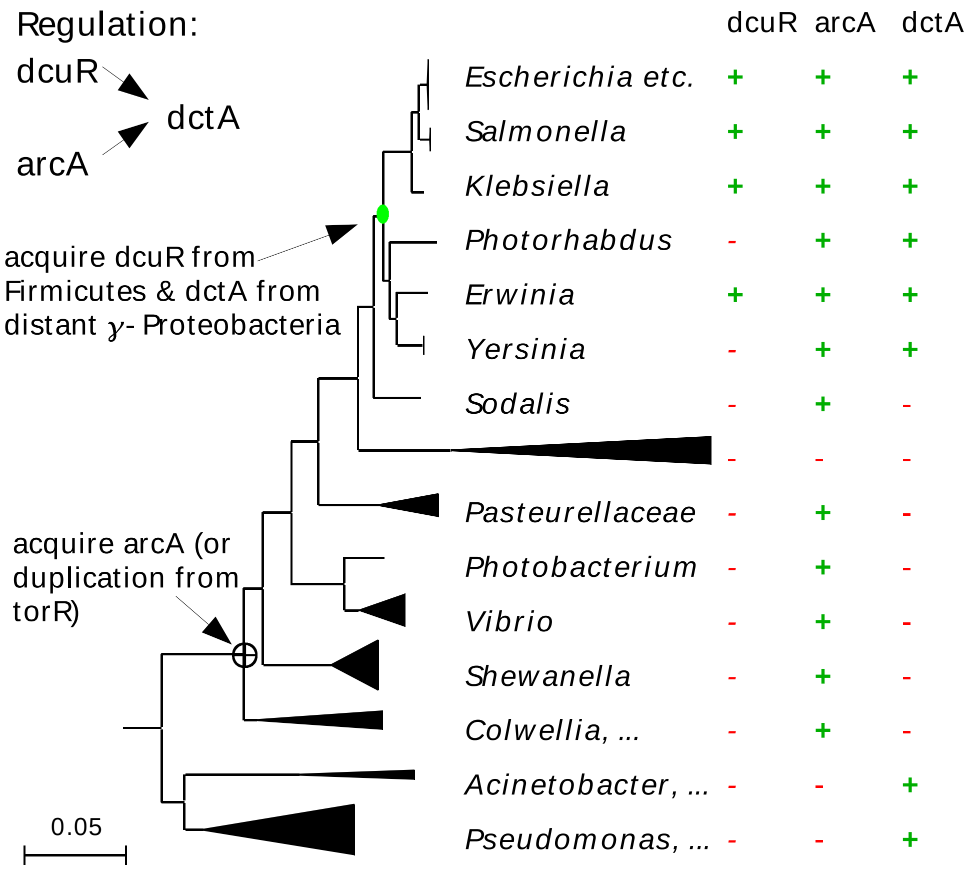

Figure 8: Complex Regulation of Horizontally Acquired Genes. HGT genes were identified by an automated presence/absence method, and the number of different regulators for each gene was taken from RegulonDB. Genes without any known regulation were not included. HGT genes tend to have more regulators than other genes ($P < 10^{-4}$, Wilcoxon rank sum test; 354 HGT genes and 998 other genes).

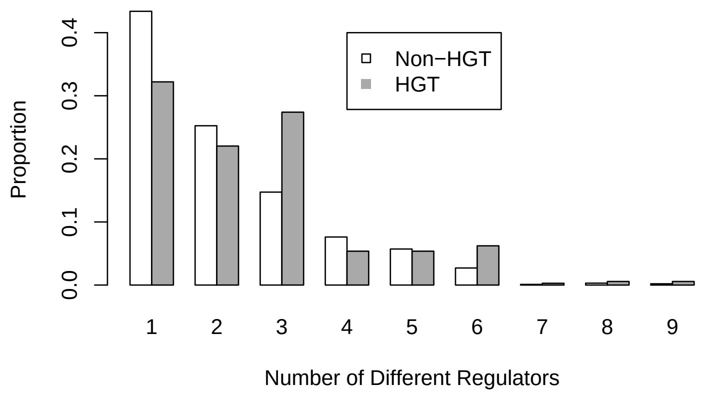

Figure 9: Automated identification of HGT genes. We examined the highest BLAST scores of homologs within groups of genomes at increasing distances from *E. coli*. If the BLAST score was substantially lower (by a factor of 1.3) in two consecutive groups relative to its best score in more distant genomes, then the gene was considered a candidate for HGT. Given such candidates, we then used a quartet test to see if the best hit from the more distant genome was actually more closely related to the *E. coli* gene than were the best hits from intermediate genomes. The quartet test confirmed HGT in 92% of these cases, and for 71% of the genes whose quartet topology indicated HGT, the topology was strongly supported ($P < 0.05$, Shimodaira-Hasegawa test in tree-puzzle (Schmidt et al., 2002)). "HPVS" refers to *Haemophilus*, *Pasteurella*, *Vibrio*, *Shewanella*, and related species.

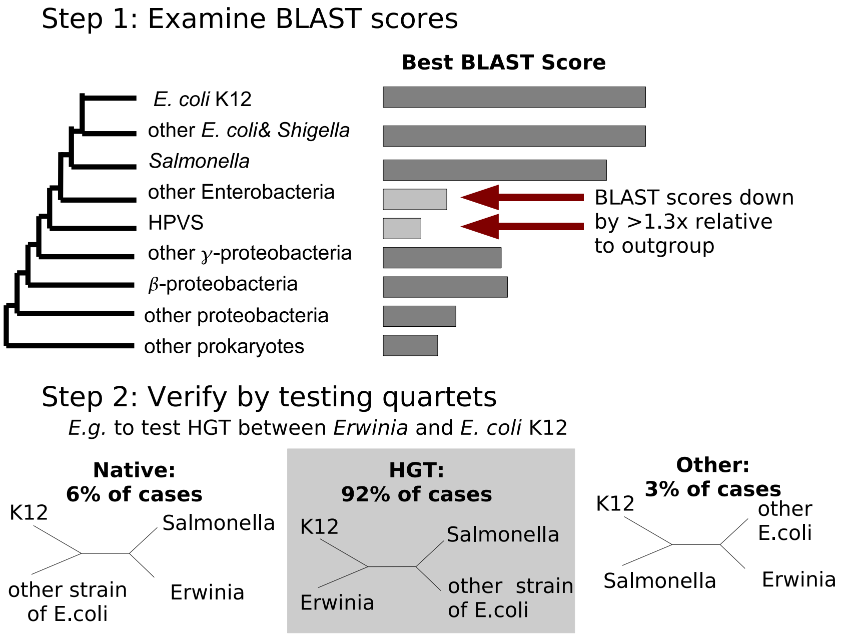

Supplementary Figure 1: A preference for HGT between related genomes. We tabulate the potential sources (as identified by best hits) of genes that were acquired by *E. coli* K12 after its divergence from other strains of *E. coli* (left panel) and the sources of genes that were acquired after the divergence of *E. coli* from *Salmonella* (right panel). We compare the distribution to that if the sources of genes were chosen at random from completely sequenced genomes. Error bars show the mean ± two standard deviations according to the binomial distribution. The most closely related groups of genomes are at the left, and the groups' names are abbreviations from Figure 9. Potential HGT events from other *E. coli* strains, from *Salmonella*, or (on the right) from other Enterobacteria are not considered because they are too close for HGT events to be identified by the automated method.

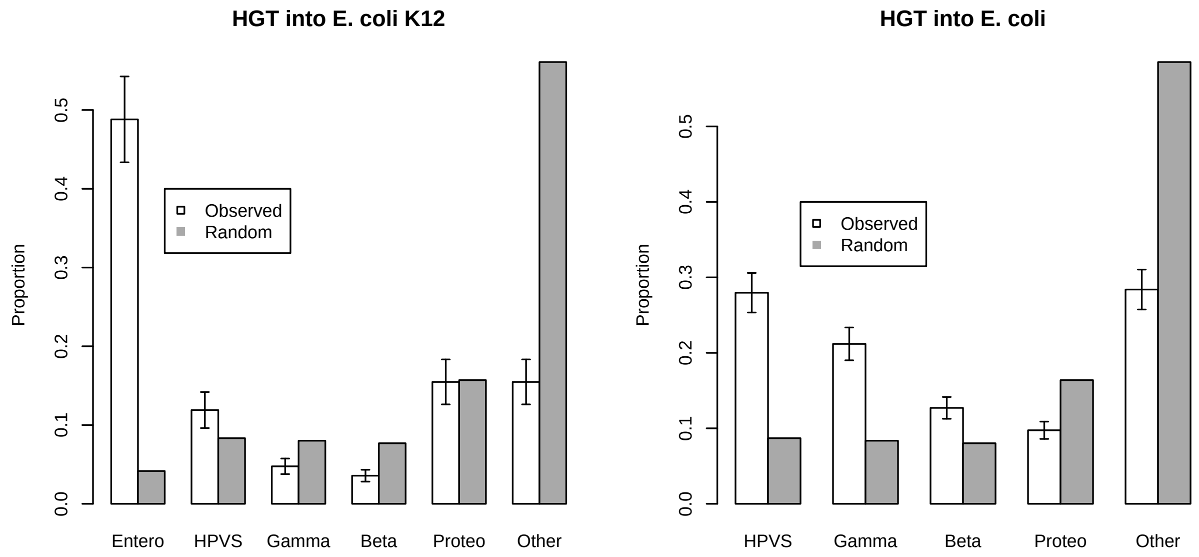

Supplementary Note 1: Detailed histories of TFs

Unless otherwise indicated, all TF functions are taken from RegulonDB (Salgado et al., 2006).

Global regulators

We examined the top 20 global regulators, as defined by the number of different genes that they are reported to regulate in RegulonDB.

Native past *Shewanella* (ancient origin in γ-Proteobacteria): arcA, crp, fis, fnr, fur, ihf (himA/himD), lrp, phoB, rpoE, rpoH, rpoN, rpoS

Native at least to *Shewanella* (previous history is unclear): cpxR, modE, narP, ntrC

HGT: flhDC, fliA, narL

Duplicated with shared regulon members ("from" gives the name of a paralog; the direction is not meaningful): hns (from stpA)

Other duplicates: none

Potential complications in the histories of "native" regulators:

- Fur is absent from the insect endosymbiont group (*Blochmannia*, *Buchnera*, *Wigglesworthia*). *Haemophilus* fur may be deeply branching (HGT), but the tree is poorly resolved because fur is so conserved. Also, *Shewanella frigidimarina* has a second (diverged copy).
- Lrp may have been transferred between *Pseudomonas* and *Bordetella.* Also, lrp appears not to be a global regulator in *Haemophilus influenzae* (D. Friedberg et al, J. Bacteriol. 183:4004-11), so even though lrp is evolutionarily ancient its role as a global regulator may have evolved more recently.
- ModE appears to be native to *Shewanella*, although there is a moderately supported grouping of *Shewanella* with Photobacterium that is incongruent the species tree. (ModE is not present in *Vibrio.*)
- NtrC (also known as glnG) seems to have been transferred from *Shewanella* to *Colwellia*, but as this does not affect the *E. coli* lineage, the gene was classified as native.
- RpoS is not present in *Sodalis*, the insect endosymbiont group, or the *Haemophilus*/*Pasteurella* group. Because these are all reduced genomes, we did not consider this to be strong evidence of an HGT event.

More detail on the instances of HGT & duplication:

- FlhDC is not a neighbor regulator, but has been transferred together with the adjacent chemotaxis genes motAB. These genes are functionally related, but are apparently regulated by fliA rather than by flhDC. FlhDC also seems to have been transferred as part of the larger flagellar system, including genes that are regulated by flhDC such as fliA and fliC, even though those genes are not nearby in *E. coli.*
- FliA is within a large cluster of flagellar genes and chemotaxis genes. Trees for fliA and for other genes in the cluster consistently group Enterobacteria with *Azotobacter* (a distantly related γ-Proteobacterium) and with β-Proteobacteria.
- Hns is reported to regulate some of the same genes as stpA, but because of the low expression of stpA in wild-type cells, this might not be physiologically relevant, and stpA may act primarily as an RNA chaperone (Zhang et al., 1996).

- NarL duplicated from narP within γ-Proteobacteria (but before the divergence of *E. coli* from *Shewanella*), and also appears to be HGT between some *Shewanella* species and a sub-group of Enterobacteria, because the narL version of the gene is absent from Vibrionaceae, from Pasteurellae, from the insect endosymbiont group that includes *Buchnera*, from *Sodalis glossinidius*, and from other Shewanellas. Because of this transfer and because narL is also heterogeneously present in more distantly related γ-Proteobacteria, narL is is classified as repeated HGT within γ-Proteobacteria. Also, narL has been transferred together with its sensor kinase narX, and probably with its regulated gene narK as well. Despite these HGT events, narP and narL are clearly ancient paralogs, and their regulons overlap considerably.

Some of these global regulators are also neighbor regulators (In Figure 6, these were included under the category of global regulators, and not under the category of neighbor regulators.)

- CpxR is a response regulator that is co-transcribed with its histidine kinase cpxA. The cxpRA operon is divergent from cpxP, which is regulated by cpxR and which inhibits cpxA (and hence cpxR, forming an autoregulatory feedback loop).
- CRP represses its own transcription and activates that of a divergent transcript that encodes a short RNA; this seems to be an antisense mechanism for CRP auto-regulation (K. Okamoto et al., J. Bacteriol. 170:5076).
- FliA regulates many flagellar genes that are colocated with fliA, including the adjacent transcript fliC.
- ModE regulates the nearby operon modABC, but because of the intervening gene b0762, which appears to be a recent insertion, modE is not, strictly speaking, a neigbhor regulator.
- NarXL is adjacent to the regulated gene narK.
- NarP is adjacent to its regulated operon napFDAGHBC-ccmADBCDEFGH.

Neighbor Regulators

After excluding 5 neighbor regulators that were also global regulators, we examined 37 of the remaining 61 neighbor regulators, primarily those that were also present in an earlier database, ColiNet.

Native: dcuR, ilvY, nhaR

Co-HGT and repeated HGT within γ-Proteobacteria ("with" gives the regulated genes that were transferred along with the TF): araC (with araB), asnC (with asnA), betI (with betT), cadC (with cadBA), cynR (with cynTSX), dsdC (with dsdXA), gntR (with gntKU), hcaR (with hcaEFCB),

lacI (with lacZ), lysR (with lysA), melR (with melAB), rtcR (with rtcAB), xapR (with xapAB), yiaJ (with yiaKLMNO-lyxK-sgbHUE)

Co-HGT only: atoC (with atoDAE), csgD (with csgBA), ebgR (with ebgAC), glcC (with glcDE-FGB), malI (with malXY), mhpR (with mhpCDF), pspF (with pspA), torR (with torCAD), treR (with treBC)

Repeated HGT only: malT, soxR

Other HGT: rpiR

ORFan: caiF, tdcR

Duplicated with shared regulon members ("from" gives the name of a paralog; the direction is not meaningful): exuR (from uxuR), galS (from galR)

Other duplicates : acrR (from envR), adiY (from envY and others), evgA (from bglJ), leuO (from ybeF or ybdO)

Complications:

- GntR and idnR duplicated within the γ-Proteobacteria, but gntR also shows a complex pattern of presence and absence, which suggests HGT of gntR along with gntKU between various γ-Proteobacteria after the duplication event. Hence gntR is classified as co-HGT/repeated-HGT above, rather than as duplicated. Nevertheless, it does reflect an older duplication, and the regulons overlap.
- The tree for ilvY suggests HGT between Enterobacteria and Shewanella, but ilvY is present in almost all of the intermediate genomes and the tree for the adjacent regulated gene ilvC shows vertical descent, so we classified ilvY as native. It is possible that ilvY was modified by homologous recombination.
- MalT has been transferred with malS, but those genes are shuffled apart in *E. coli*, and so it is not classified as co-HGT.

We also classified the co-HGT neighbor regulators by their action:

- Activators: atoC, cadC, csgD, glcC, mhpR, pspF, xapR
- Repressors: betI, ebgR, gntR, lacI, malI, rtcR, treR, yiaJ
- Both: araC, asnC, cynR, dsdC, hcaR, lysR, melR, torR

Thus, we had roughly equal numbers of each class amongst the co-HGT neighbor regulators (7, 8, 8). Similarly, when considering all regulators, we had roughly equal numbers (50, 51, 58).

Other Characterized Regulators

We examined a random sample of 25 out of 78 characterized non-global non-neighbor regulators. Because HU is a heterodimer (hupA/hupB) of two genes with different histories, the actual number of genes tabulated is 26 out of 79.

Native: birA, fabR, dnaA, glpR, hupB, nsrR, oxyR, pdhR

HGT: alpA, cueR, cytR, deoR, gutM, hipB, hupA, idnR, kdgR, nac, rcsB

ORFan: hycA

Duplicated with shared regulon members: cspA (from cspBEG), galR (from galS), marA (from soxS or rob)

Other duplicates: appY (from gadW and others), iclR (from allR), ydeO (from gadW or appY)

Comments:

- As explained in the text, alpA, gutM, and hipB are co-HGT with nearby genes. Also, RegulonDB and ColiNet report that alpA regulates slp, but the original publication (Trempy *et al.*, 1994) gives the sequence of the regulated gene (which they term slpA), and the sequence matches that of intA (also known as b2622), rather than that of slp. IntA is separated from alpA by a single gene, so alpA is not classified as a neighbor regulator.

- AlpA, cueR, deoR, and kdgR show repeated HGT within γ-Proteobacteria.

- CueR is adjacent to its regulated gene copA in other Enterobacteria, but in *E. coli* these are separated by two other genes. This suggests that cueR was originally a neighbor regulator.

- FabR, which regulates the synthesis of unsaturated fatty acids, is adjacent to a fatty acid desaturase in Shewanella. Although that enzyme is not conserved in *E. coli*, this suggests that fabR may have originally been a neighbor regulator.

- The history of hupA is unclear. It is clearly related to hupB, but intermediate relatives are from distant taxa. Furthermore, hupB seems to lack close paralogs in distant γ-Proteobacteria, and Shewanellas seem to have a different paralog of hupB. Hence, hupA seems to have arisen recently, perhaps by acquisition from distant organisms, and so it is classified as HGT.

- IdnR is not listed as a neighbor regulator because it is co-transcribed with its regulated gene idnT. These genes were duplicated together (from gntR/gntT).

- PdhR is an ancient paralog of lldR (also known as lctR), with which it shares regulation. Because this event occurred before the divergence of *E. coli* from *Shewanella*, it was classified as native.

Putative (Uncharacterized) Regulators

We examined a random sample of 20 out of 96 putative regulators (predicted regulators that are not reported to regulate any genes in RegulonDB 5.6). We verified that the putative regulators were predicted to contain DNA binding domains and that these DNA binding domains were not predicted to have other non-regulatory functions. As of May 31, 2007, EcoCyc does not report any function for these genes. A few of them are in operons with other characterized genes and hence have been given names.

Native: none

co-HGT: b2382 (with b2383), ybcM (with ybcL), ybhD (with ybhH), ybiH (with b0795), yfeG (with eutBC), yfhA (with yfhKG) ygeV (with ygeWXYZ), yneJ (with b1525), ynfL (with ynfM)

Other HGT: b1439, b1506, b1770, yahA, yfhH, yidL, yihL, ykgD

Duplicated: yijO (from b2382), ykgK (from yqeH)

ORFan: b1685

Of these HGT genes, we classified 7 as repeated HGT within γ-Proteobacteria: b1439, yahA, yfhA, yfhH, yihL, ykgD yneJ

Supplementary Note 2: Evolutionary ages of paralogous regulatory interactions – are they conserved from a common ancestor?

We analyzed a random sample of the paralogous regulatory interactions reported by Teichmann and Babu (2004).

TF regulates two paralogous genes

Teichmann and Babu (2004) propose that if a TF regulates two paralogous genes, then this evolved by the duplication of the regulated gene together with its promoter region. We examined 10 examples at random (from "Model 1" in their supplementary material, http://www.mrc-lmb.cam.ac.uk/genomes/madanm/net_evol/ec_m1_272.txt) and classified them as follows.

Paralogous genes diverged before the regulatory interaction evolved (6):

- arcA → aceB & glcB: glcB was acquired after the divergence of *E. coli* from *Salmonella*, and glcB has close relatives in diverse bacteria including Bacilli. arcA has evolutionary orthologs only within γ-Proteobacteria (e.g. it has a paralog torR within this group). This suggests that glcB was acquired from a bacterium that did not contain arcA and hence that this regulation evolved after the divergence of aceB from glcB.
- arcA → cyoC & sdhC & sdhD: sdhC and sdhD are ancient enzymes in the TCA cycle, and hence this divergence presumably predates arcA. cyoC is also present in diverse organisms.
- crp → rhaA & yiaR: crp is unique to Proteobacteria, while rhaA and yiaR both show recent HGT with other phyla.
- fis → leuP & many other tRNAs: fis is unique to Proteobacteria, and distant relatives in other Proteobacteria have other functions (e.g., ntrC in Rhizobium). It is possible that some of these regulated tRNAs evolved by duplication (e.g., S. Giroux and R. Cedergen, J. Bacteriology 171:6446-54), but presumably most of these tRNAs diverged from each other before the Proteobacteria arose.
- himA → hycG & nuoB: himA is well-conserved within Proteobacteria, but it does not have orthologs in most other phyla (e.g., Firmicutes and Cyanobacteria, although these do contain homologous HU-like proteins). Thus, himA is ancient, but probably not older than the Proteobacteria. hycG is a recent paralog of hyfI and their closest relative is from Firmicutes (e.g., *Thermoanaerobacter tengcongensis*), so the hycG/hyfI ancestor was probably acquired from bacteria that did not have himA.

- narL → fdnI & frdC & frdD: narL is relatively recent HGT within the γ-Proteobacteria, and furthermore is a paralog of narP within γ-Proteobacteria. Only narL is reported to regulate frdABCD, while fdnGHI has sites for both as well as some narL-only sites. frdC and frdD are very distantly related (the homology is not detectable by BLAST and they are assigned to different PFams). frdCD is native to Shewanella and then has homologs in Chromobacterium and Mycobacteria, which do not contain narL/narP, which suggests that its regulation arose after it was acquired.

Operon complications (1):

- himA (IHF) → tdcE & pflB: tdcE and pflB are relatively recent paralogs, and IHF is well conserved within Proteobacteria, so this could be an ancestral relationship. However, these genes are in operons with non-paralogous genes, and neither tdcE nor pflB is the first gene in their operon, so this cannot have evolved by simply duplicating a gene together with its promoter region.

Unclear (1):

- cysB → cysH & cysM: cysH and cysM are ancient paralogs of each other and also of cysK. cysB has a paralog cbl (also known as metC) within β, γ-Proteobacteria, so it is possible that their common ancestor regulated cysH and cysM. However, more distant relatives of cysB are found in diverse bacteria and probably have diverse functions. For example, a subfamily found in Xanthomonas campestris (YP_241927) and other γ-Proteobacteria has been transferred together with genes for leucine synthesis. Thus, we doubt whether the regulatory role of cysB/cbl is as old as the divergence of cysH from cysM.

Evolution by duplication (1):

- fliA → tarT, tap, tsr. tar/tsr are recent paralogs, and fliA is widely conserved in γ-Proteobacteria.

Other (1):

- purR → gcvP: The only paralog of gcvP we identified was rtcB, which is not regulated by purR, so we do not know why this was included in Teichmann & Babu's analysis.

Paralogous TFs regulate the same gene

Teichmann and Babu (2004) propose that if paralogous TFs regulate the same gene, then this evolved by the duplication of the TF. We examined 10 examples at random (from "Model 2" in their supplementary material, http://www.mrc-lmb.cam.ac.uk/genomes/madanm/net_evol/ec_m2_128.txt) and classified them as follows.

TF duplication predates acquisition of regulated gene (6):

- arcA & dcuR → dctA: As discussed above, arcA is orthologous within γ-Proteobacteria. dcuR, which is also known as yjdG, is present in Enterobacteria but not in more distantly related γ-Proteobacteria and seems to have been acquired by HGT, perhaps from Firmicutes.
- arcA & narL → nuoL: nuoL is in Enterobacteria but not in other relatives and seems to have been acquired by HGT. Both arcA and narL have older origins within the γ-Proteobacteria.
- arcA & narL → nuoN: As with nuoL, nuoN is in Enterobacteria but not in most other relatives.
- cbl & cysB → tauD: cysB and cbl are paralogs within the β,γ-Proteobacteria, while tauD is present in Enterobacteria such as Yersinia but not in more distant γ-Proteobacteria.
- crp & fnr → ansB: ansB was acquired by HGT, probably after the divergence of *E. coli* from *Vibrio* species, and has close homologs in other phyla, while crp and fnr are ancient native genes within Proteobacteria.
- lysR & tdcA → tdcA: lysR has a complex history of HGT, as does tdcA, and these genes are distantly related.

Unclear (4):

- crp & fnr → sucA: Both regulators and the regulated gene are ancient native genes within Proteobacteria, so it is hard to determine if the duplication predates the regulation or not.
- crp & fnr → sucB: Both regulators and the regulated gene are ancient native genes within Proteobacteria, so it is hard to determine if the duplication predates the regulation or not.
- crp & fnr → tdcA: The regulation of tdcA by fnr seems to be indirect (Chattopadhyay et al., J. Bacteriol. 179:4868-73). In any case, crp and fnr are ancient paralogs and are both highly conserved within γ-Proteobacteria. tdcA is a recent paralog of ydaK and has a complex history of HGT before that. Because many of these homologs are in β,γ-Proteobacteria, we cannot rule out the possibility that tdcA was regulated by crp and fnr before the acquisition.
- crp & fnr → tdcG: tdcG is in the same operon as tdcA and has the same history.

Paralogous TFs regulate paralogous genes

Teichmann and Babu (2004) propose that if paralogous TFs regulate paralogous genes, then this evolved by the duplication of both the TF and the regulated genes. We examined 10 examples at random (taken from "Model 3" in their supplementary material, http://www.mrc-lmb.cam.ac.uk/genomes/madanm/net_evol/ec_m3_74.txt) and classified them as follows.

Autoregulation of distantly related TFs (4):

- arsR → arsR & marR → marR.
- betI → betI & uidR → uidR.
- asnC → asnC & lrp → lrp.
- galS → galS & idnR → idnR.

(Because auto-regulation is common for all types of transcription factors, it is not surprising that distantly related pairs of TFs are found in which both members of the pair regulate their own transcription. Hence, there is no reason to expect that this reflects conserved regulation from a common ancestor.)

Unclear (2):

- evgA → ompC & ompR → fadL, ompC, ompF & phoB → phoE : These two-component systems have different functions and have closer paralogs: evgA has closer paralogs bglJ, dctR, and rcsA; phoB has closer paralogs baeR and creB; ompR has closer paralogs cpxR and torR. This suggests that the regulatory cross-talk between these two-component systems arose after the duplication events.
- fur → fepC & fhuC & zur → znuC. (zur is also known as yjbK and znuC is also known as yebM.) zur and fur are ancient paralogs. fepC, znuC, and fhuC all show evidence for recent HGT. However, because zur is adjacent to znuC in some distant bacteria, it is possible that the two have co-evolved and been transferred together since the divergence.

Other (4):

- gcvA → gcvP: We did not find regulatory relationships between paralogs for gcvA (cbl, cynR, cysB, dsdC, hcaR, ilvY, lysR, metR, nac, nhaR, oxyR, tdcA, xapR) and gcvP (rtcB), so we do not know why this was included in Teichmann & Babu's analysis.

- marR → nfo: We did not find regulatory relationships between paralogs for marR (arsR,gatR_2) and nfo (rhaA,uxuA,xylA,yiaR), so we do not know why this was included in Teichmann & Babu's analysis.
- metR → glyA: We did not find regulatory relationships between paralogs for metR (cbl, cynR, cysB, dsdC, gcvA, hcaR, ilvY, lysR, nac, nhaR, oxyR, tdcA, xapR) and glyA (argD, bioA, bioF, metC, kbl, malY, tnaA, tyrB), so we do not know why this was included in Teichmann & Babu's analysis.
- yiaJ → yiaQ: We did not find any regulatory relationships between paralogs for yiaJ (iclR,mhpR) and yiaQ (trpC), so we do not know why this was included in Teichmann & Babu's analysis.

Supplementary Note 3: Evolutionary ages of regulatory interactions shared by close paralogs

We examined all regulatory interactions in RegulonDB 5.6 that are shared between close paralogs (defined by a bit-score above 30% of self-score). We found 212 cases where paralogous TFs regulated the same operon, 290 cases where paralogous genes were regulated by the same TF, and 54 cases where paralogous TFs regulated paralogous genes.

Paralogous TFs regulate the same operon

By eliminating mirror symmetry, 212 cases were reduced to 106, and which further reduced to 35 operons, which are listed below. Note that autoregulated genes are included in the operons below, but are not included in the count of 106 cases or genes.

Convergent evolution (10 regulated operons, 32 regulated genes):

**TF1**	**TF2**	**Regulated Operon**
Regulated genes acquired after the duplication; TFs bind different sites		
arcA	torR	gadAX (arcA site is predicted to be far upstream (Salmon et al., 2005))
baeR	cpxR	acrD
baeR	cpxR	mdtABCD-baeSR
cbl	cysB	ssuEADCB
Regulated genes acquired after the duplication; sites not known		
cbl	cysB	tauABCD
narL	narP	fdhF
Regulated genes acquired after the duplication, but TFs bind the same site		
gntR	idnR	idnDOTR
narL	narP	fdnGHI
narL	narP	hcp-hcr
narL	narP	hyaABCDEF

Unclear (10 regulated operons, 32 regulated genes):

TF1	TF2	Regulated Operon
Relative ages unclear, and the TFs bind the same site		
cpxR	ompR	csgDEFG
cpxR	ompR	ompC
cpxR	ompR	ompF
fhlA	hyfR	hyfABCDEFGHIJR-focB
gntR	idnR	idnK
marA	soxS	acrAB
marA	soxS	marRAB
narL	narP	norVW
narL	narP	nrfABCDEFG
TF duplication is younger than the regulated gene, but the sites are different		
marA	soxS	fumC

Comments: ompC and ompF have a complex history of additional duplication events after the original divergence. Some of the extra paralogs have similar regulation, but none of them are regulated by both cpxR and ompR. Nevertheless, the regulation of these genes could have been conserved, while that of the other paralogs diverged.

Evolution by duplication (15 operons, 42 regulated genes):

TF1	TF2	Regulated Operon
TF duplication is probably younger than the regulated gene, and the site is shared		
exuR	uxuR	uxuAB
galR	galS	galETKM
galR	galS	galP
galR	galS	mglBAC
gntR	idnR	gntKU
marA	soxS	fpr
marA	soxS	inaA
marA	soxS	nfo
marA	soxS	poxB
marA	soxS	pqiAB
marA	soxS	sodA
marA	soxS	zwf
mlc	nagC	manXYZ
narL	narP	napFDAGHBC-ccmABCDEFGH
narL	narP	nirBDC-cysG

Comments: In RegulonDB, poxB is listed as being in an operon with ltaE and ybjT, but only poxB is listed as being regulated by marA or soxS. Similarly, gntKU are listed as being in an operon with gntR, but only gntKU are regulated by gntR and idnR, because of an internal promoter.

Paralogous Genes Are Regulated by the Same TF

By eliminating mirror symmetry, 290 paralogous gene-TF interactions reduced to 145 cases. In only 62 of these cases were the first two genes in the operon close homologs, so that the operon structures were consistent with evolution by duplication. We classified those and list them below. Usually, not all the genes in the operons are paralogs – the paralogs are in bold. We also examined some of the cases where the operon structures were not consistent with evolution by duplication, and confirmed that these usually involved ancient duplications or movement of a gene to a preexisting operon (data not shown).

Evolution by duplication (17 duplication events accounting for 38 cases):

**Operon**	**Operon**	**TF(s)**
**araE**	**galP**	crp
**argF**	**argI**	argR
cyd**AB**	app**CB**A	arcA
dms**ABC**	ynf**EFGH**-dmsD	fnr
**fimB**	**fimE**	hns
**fldA**-fur	**fldB**	soxS
gad**B**C	gad**A**X	gadE, crp, hns, rpoS, yhiW, yhiX
gnt**K**U	**idnK**	crp, gntR, idnR
liv**K**HMGF	**livJ**	lrp
**nmpC**	**ompC**	himA, ompR
**nmpC**	**ompF**	crp, himA, ompR
**ompF**	**ompC**	cpxR, envY, himA, lrp, ompR
**tar**-tap-cheRBYZ	tar-**tap**-cheRBYZ	fliA, fnr
**tar-tap**-cheRBYZ	**tsr**	fliA
**trg**	**tar-tap**-cheRBYZ	fliA
**trg**	**tsr**	fliA
ynf**E**FGH-dmsD	ynfE**F**GH-dmsD	fnr

Unclear cases involving long-diverged genes – 7 putative duplication events accounting for 13 cases:

**Operon**	**Operon**	**TF(s)**
**argT**-hisJQMP	argT-his**J**QMP	glnG, rpoN
**astC**ADBE	**argD**	argR
**gapA**	**epd**-pgk-fbaA	crp
ibp**A**B	ibpA**B**	rpoH
mdtA**B**CD-baeSR	mdtAB**C**D-baeSR	baeR, cpxR
oppABC**D**F	oppABCD**F**	arcA, lrp, modE
**sodB**	**sodA**	crp, fur, himA

Comments on unclear cases: mdtB/mdtC and oppD/oppF might have arisen from an ancient tandem duplication event, followed by evolution of new regulation for the operon in the *E. coli* lineage.

Covergent evolution, where one of the regulated genes was acquired after the duplication – 7 putative duplication events accounting for 11 cases:

**Operon**	**Operon**	**TF(s)**
**aroG**	**aroF**-tyrA	tyrR
dsd**X**A	**gntT**	crp
**gntT**	**gntP**	crp
gudP**X**D	gudPX**D**	yaeG
gud**P**XD	gar**P**LRK-rnpB	yaeG
**hupB**	**hupA**	crp, fis
hya**AB**CDEF	hyb**O**AB**C**DEFG	arcA, narL

Comments on the convergent cases: The proximity of gudX and gudD suggests a tandem duplication, but the phylogenetic tree implies otherwise – gudD is present only in Enterobacteria, and it branches more deeply than gudY from other lineages.

Paralogous Genes Are Regulated by Paralogous TFs

By eliminating mirror symmetry, 54 cases were reduced to 27, which are listed below:

Duplication of both TF and regulated gene(s) – 4 cases:

$\mathbf{TF_1}$	$\mathbf{Operon_1}$	$\mathbf{TF_2}$	$\mathbf{Operon_2}$
gatR	gatY**Z**ABCD	agaR	aga**Z**VWA
gatR	gat**Y**ZABCD	agaR	agaS**Y**BCDI
gntR	**idnK**	idnR	gnt**K**U
gntR	gnt**K**U	idnR	**idnK**

Convergent evolution, with complex HGT of regulated genes – two putative duplication events, 8 cases:

$\mathbf{TF_1}$	$\mathbf{Operon_1}$	$\mathbf{TF_2}$	$\mathbf{Operon_2}$
narL	hyb**O**AB**C**DEFG	narP	hya**AB**CDEF
hyfR	hyf**A**B**C**DEF**GHIJ**R-focB	fhlA	hycA**B**C**DEFGH**I

Convergent evolution – TF duplication precedes that of regulated genes – 15 cases:

**TF$_1$**	**Operon$_1$**	**TF$_2$**	**Operon$_2$**
baeR	mdtA**B**CD-baeSR	cpxR	mdtAB**C**D-baeSR
baeR	mdtAB**C**D-baeSR	cpxR	mdtA**B**CD-baeSR
cpxR	**ompC**	ompR	**nmpC**
cpxR	**ompC**	ompR	**ompF**
cpxR	**ompF**	ompR	**nmpC**
cpxR	**ompF**	ompR	**ompC**
gntR	**gntT**	idnR	gntK**U**
gntR	idnDO**T**R	idnR	gntK**U**
gntR	**gntT**	idnR	idnDO**T**R
gntR	gntK**U**	idnR	idnDO**T**R
phoB	**phoE**	ompR	**nmpC**
phoB	**phoE**	ompR	**ompC**
phoB	**phoE**	ompR	**ompF**
phoB	**phoE**	cpxR	**ompC**
phoB	**phoE**	cpxR	**ompF**

Comments: the similar regulation of ompC, ompF, and nmpC probably reflects duplication of the genes (but not of the TFs). gatR and agaR appear to have co-evolved with their regulated operons since their regulated operons, despite more recent HGT events and changes in operon structure.
